# Cellular Control of Cortical Actin Nucleation

**DOI:** 10.1016/j.cub.2014.05.069

**Published:** 2014-07-21

**Authors:** Miia Bovellan, Yves Romeo, Maté Biro, Annett Boden, Priyamvada Chugh, Amina Yonis, Malti Vaghela, Marco Fritzsche, Dale Moulding, Richard Thorogate, Antoine Jégou, Adrian J. Thrasher, Guillaume Romet-Lemonne, Philippe P. Roux, Ewa K. Paluch, Guillaume Charras

**Affiliations:** 1 London Centre for Nanotechnology, University College London, London WC1H 0AH, UK; 2 Department of Cell and Developmental Biology, University College London, London WC1E 6BT, UK; 3 Institute for Research in Immunology and Cancer, Université de Montréal, Montréal, QC H3N 3J7, Canada; 4 Department of Physics and Astronomy, University College London, London WC1E 6BT, UK; 5 Max Planck Institute of Molecular Cell Biology and Genetics, Dresden 01307, Germany; 6 International Institute of Molecular and Cell Biology, Warsaw 02-109, Poland; 7 Medical Research Council Laboratory for Molecular Cell Biology, University College London, London WC1E 6BT, UK; 8 Institute of Child Health, University College London, London WC1N 1EH, UK; 9 Laboratoire d’Enzymologie et Biochimie Structurales, CNRS, 91198 Gif-sur-Yvette, France

## Abstract

The contractile actin cortex is a thin layer of actin, myosin, and actin-binding proteins that subtends the membrane of animal cells. The cortex is the main determinant of cell shape and plays a fundamental role in cell division [1–3], migration [4], and tissue morphogenesis [5]. For example, cortex contractility plays a crucial role in amoeboid migration of metastatic cells [6] and during division, where its misregulation can lead to aneuploidy [7]. Despite its importance, our knowledge of the cortex is poor, and even the proteins nucleating it remain unknown, though a number of candidates have been proposed based on indirect evidence [8–15]. Here, we used two independent approaches to identify cortical actin nucleators: a proteomic analysis using cortex-rich isolated blebs, and a localization/small hairpin RNA (shRNA) screen searching for phenotypes with a weakened cortex or altered contractility. This unbiased study revealed that two proteins generated the majority of cortical actin: the formin mDia1 and the Arp2/3 complex. Each nucleator contributed a similar amount of F-actin to the cortex but had very different accumulation kinetics. Electron microscopy examination revealed that each nucleator affected cortical network architecture differently. mDia1 depletion led to failure in division, but Arp2/3 depletion did not. Interestingly, despite not affecting division on its own, Arp2/3 inhibition potentiated the effect of mDia1 depletion. Our findings indicate that the bulk of the actin cortex is nucleated by mDia1 and Arp2/3 and suggest a mechanism for rapid fine-tuning of cortex structure and mechanics by adjusting the relative contribution of each nucleator.

## Highlights

• The Arp2/3 complex and the formin mDia1 nucleate the bulk of cortical F-actin
• Nucleator depletion alters the cortex ultrastructural organization
• Actin nucleated by mDia1 accumulates faster than actin nucleated by Arp2/3
• mDia1 depletion leads to mitosis failure, an effect potentiated by Arp2/3 inhibition

## Teaser

Bovellan et al. show that two actin nucleators, the formin mDia1 and the Arp2/3 complex, generate the bulk of the submembranous cortical F-actin. These nucleators have very different actin accumulation kinetics and play distinct roles in cortical actin organization, and their depletion differentially affects progression through the cell cycle.

## Results and Discussion

Here, we took an unbiased approach to study cortical actin nucleation. We used natural and induced cellular blebs as tools; expanding blebs are initially devoid of F-actin and progressively reassemble a contractile cortex prior to retraction [16], making them an ideal model to study de novo cortex assembly. Thus, we reasoned that the proteins necessary for the regrowth of cortical actin should be present in blebs. Cortex assembly could occur via elongation of F-actin seeds or mediated by nucleators. We first examined several seed elongation cortical growth mechanisms and concluded that these were not supported by experimental evidence (see Figure S1 available online). Therefore, we investigated the role of actin nucleators in cortex assembly using two independent unbiased approaches.

**Figure 1:**
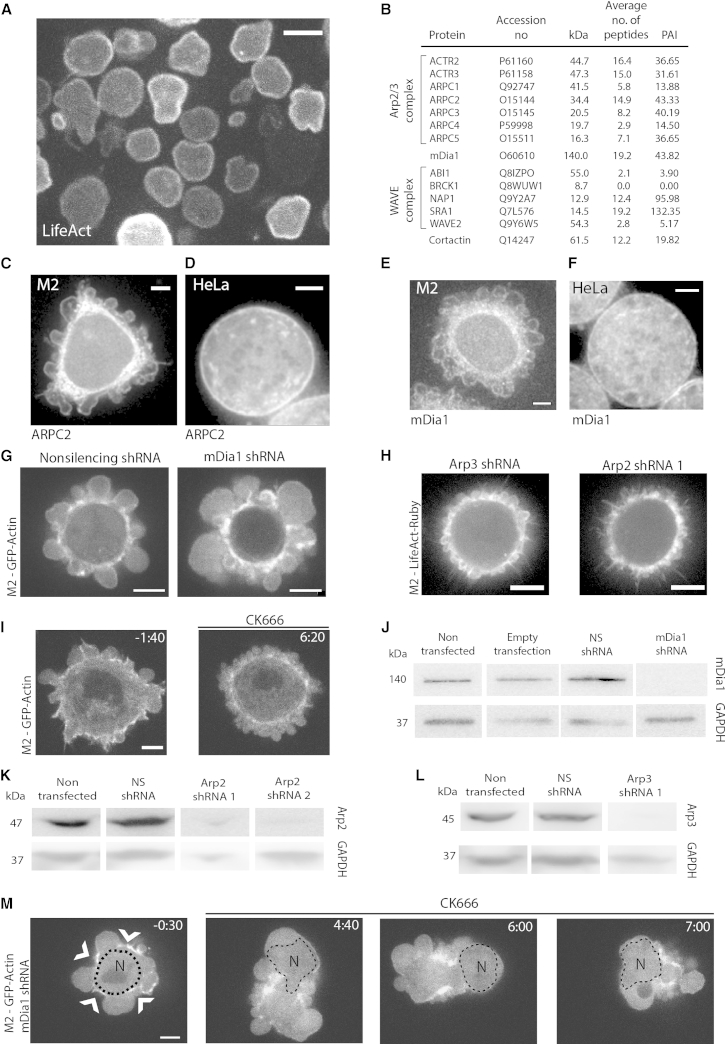
Perturbation of the Activity of the Formin mDia1 and the Arp2/3 Complex Leads to Changes in Cell Morphology In all panels, images are single confocal planes, and scale bars represent 5 μm unless otherwise indicated. (A) Blebs separated from M2 melanoma cells stably expressing the F-actin reporter LifeAct-Ruby. (B) Actin nucleators and nucleation-promoting factors detected in the detergent-insoluble fraction of separated blebs. Subunits of the same complex are displayed together. Protein isoforms are grouped together (see Supplemental Experimental Procedures). Detected peptide numbers are averaged over three separate experiments. When this was larger than 3, the protein was detected with high certainty. Protein abundance index (PAI) was calculated based on spectral counts (see Supplemental Experimental Procedures). (C–F) Immunofluorescence image of an M2 blebbing cell stained with anti-ARPC2 (C), a mitotic HeLa cell stained with anti-ARPC2 (D), an M2 blebbing cell stained with anti-mDia1 (E), and a mitotic HeLa cell stained with anti-mDia1 (F). (G) Live confocal microscopy images of M2 melanoma cells stably expressing EGFP-actin and stably transfected with nonsilencing shRNA (NS, left) or shRNA targeting mDia1 (right). See Movie S1. (H) Live confocal microscopy images of M2 melanoma cells stably expressing LifeAct-Ruby and stably transfected with shRNA targeting Arp2 (Arp2 shRNA 1, left) or Arp3 (Arp3 shRNA 1, right). See Movie S2. (I) M2 cells stably expressing GFP-actin treated with CK666. 100 μM CK666 was added at time point 0:00. Time is in min:s. Intensity scales are kept constant between images. Scale bar represents 3 μm. (J) Immunoblot of M2 cells stably transfected with NS or mDia1 shRNA 1 probed with anti-mDia1 and anti-GAPDH. (K) Immunoblot of M2 cells stably transfected with NS, Arp2 shRNA 1, or Arp2 shRNA 2 probed with anti-Arp2 and anti-GAPDH. (L) Immunoblot of M2 cells stably transfected with NS or Arp3 shRNA 1 probed with anti-Arp3 and anti-GAPDH. (M) Live imaging of an mDia1 stably depleted M2 cell treated with CK666. The cell also stably expressed GFP-actin. 100 μM CK666 was added at time point 0:00. The nucleus (N) is outlined with the dashed line. Time is in min:s. See Movie S3. See also Figure S1.

First, we used proteomics on isolated cortices to identify the actin nucleators present in the cortex. To this aim, we separated blebs from constitutively blebbing M2 melanoma cells by mechanical shearing as previously described [17], a procedure that allows isolation of dynamic actin cortices (Figure 1A). We investigated the presence of actin nucleators in the actin-rich detergent-insoluble fraction of isolated blebs using mass spectrometry analysis. We detected the presence of only two actin nucleators: the formin mDia1 and the Arp2/3 complex (Figure 1B), consistent with some reports [11, 13] but in contradiction with others [8–10, 12]. All seven subunits of the Arp2/3 complex were detected along with the Arp2/3 nucleation-promoting factors cortactin and two subunits of the WAVE complex (SRA1 and NAP1).

To verify these results using an independent approach, we undertook a screen based on mRNA expression profiles, protein localization, and small hairpin RNA (shRNA) depletion. First, we determined which nucleators were expressed in both M2 and HeLa cells and localized to their cortex, reasoning that basic mechanisms of cortex nucleation should be conserved across cell types. Quantitative PCR revealed that ten nucleators were expressed in both cell lines: the Arp2/3 complex subunits *ACTR2* and *ACTR3*; the formins *DAAM1*, *DIAPH1*, *DIAPH3*, *FHOD1*, *FMNL1*, *INF1*, and *INF2*; and *SPIRE1* and *SPIRE2* (Figure S2A). To distinguish potential cortical actin nucleators, we examined the localization of the expressed nucleators in M2 cells using imaging assays, reasoning that proteins clearly localized to the cell periphery were good candidates for initiators of cortex growth, whereas nucleators strongly enriched in other locations were likely fulfilling other functions. Of the expressed nucleators, only Daam1, Fhod1, mDia1, and the Arp2/3 complex were found to localize to the cell periphery (Figures 1C–1F and S2B–S2I). Daam1 was present in the membrane of both expanding and retracting blebs (Figure S2B). Fhod1 accumulated at the actin cortex late in bleb retraction (Figure S2C). Immunostaining for mDia1 and the ARPC2 subunit of the Arp2/3 complex revealed clear localization to the cortex of M2 cell blebs (Figures 1C and 1E) and mitotic HeLa cells (Figures 1D and 1F).

Having narrowed down potential candidates to mDia1, Fhod1, Daam1, and the Arp2/3 complex, we depleted these proteins with shRNA and used the size of blebs in M2 cells as reporters of perturbations to cortical actin. We reasoned that silencing of a cortical nucleator might lead to either larger blebs due to weakening of the cortex as a result of lower F-actin polymerization (as observed upon cytochalasin D treatment, Figure S2J) or smaller blebs due to disruption of contractility because of perturbation of cortical F-actin organization [18]. Stable knockdown of Fhod1 did not lead to significant changes in bleb size in M2 cells (p = 0.5 compared to nonsilencing shRNA [NS]; Figures S2K, S2M, S2O, and S2Q; Table S1). Similar results were observed with transient transfection with two other shRNAs (∼50% mRNA depletion; data not shown). Stable depletion of Daam1 led to significant changes in bleb size distribution in one out of two knockdown lines (two different shRNAs, p = 0.03 and p < 0.001, respectively, compared to NS; Figures S2K, S2L, S2P, and S2Q; Table S1). Stable knockdown of mDia1 led to cells with significantly larger blebs (Figures 1G, 1J, S2K, and S2Q; Table S1; Movie S1). A similar phenotype was observed with transient transfection for three other shRNAs (Figure S2N). Stable knockdown of the Arp3 or Arp2 subunits of the Arp2/3 complex did not inhibit blebbing (consistent with [19]) but led to cells with significantly smaller blebs (Figures 1H, 1K, 1L, S2K, and S2Q; Movie S2; Table S1). When we acutely perturbed Arp2/3 activity using the selective inhibitor CK666 [20], we observed a phenotype with small blebs in ∼60% of cells (Figure 1I), consistent with depletion experiments, thus suggesting that CK666 is a good substitute for gene depletion. Finally, combined perturbation of mDia1 and Arp2/3 by gene depletion and CK666 treatment led to two distinct phenotypes: 34% of cells became amorphous and retained only a few discernible foci of cortical actin, while 30% of cells formed very large blebs (Figure 1M; t > 0 s; Movie S3). Together, these data suggest that in M2 cells, mDia1, Arp2/3, and Daam1 play an important role in the control of bleb size and may thus be involved in actin cortex nucleation.

**Figure 2:**
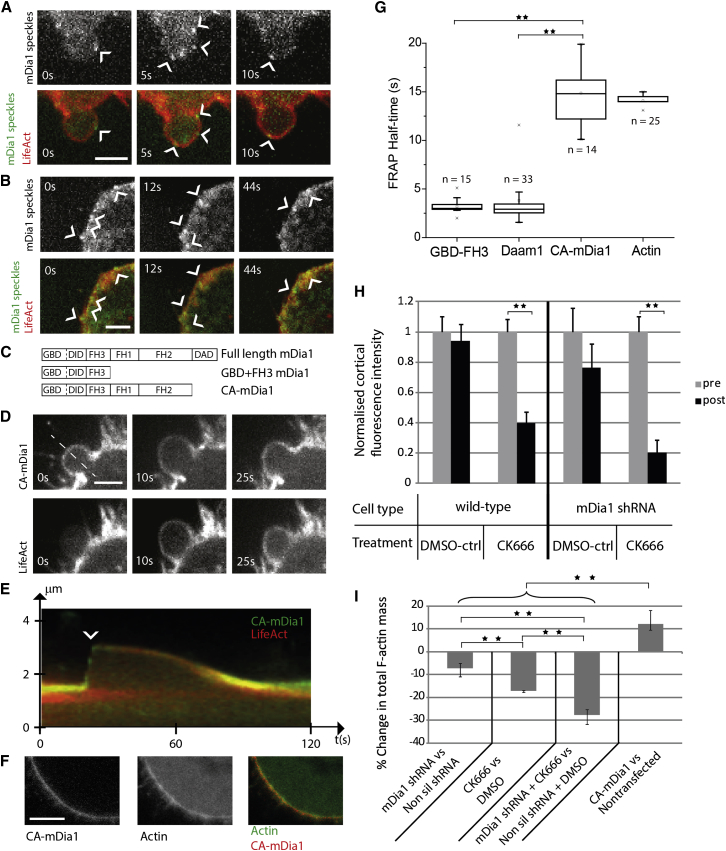
mDia1 Binds to the Cell Cortex and Contributes to Cortical F-Actin Levels along with the Arp2/3 Complex (A) Single-molecule imaging of full-length GFP-mDia1 (green in lower panels) in M2 cells stably expressing LifeAct-Ruby (red in lower panels). Arrowheads indicate mDia1 speckles. Scale bar represents 3 μm. (B) Single-molecule imaging of full-length GFP-mDia1 (green in lower panels) in mitotic HeLa cells stably expressing LifeAct-Ruby (red). Arrowheads indicate mDia1 speckles. Scale bar represents 3 μm. (C) mDia1 constructs used in this study. A full-length protein, a membrane-targeted deletion mutant with no actin nucleation activity comprising only the GBD and FH3 domains (GBD+FH3), and a constitutively active mutant with a deletion of the DAD domain preventing autoinhibition (CA-mDia1) are shown. (D) Localization of GFP-CA-mDia1 at different stages of the bleb life cycle in M2 cells stably expressing LifeAct-Ruby. At the onset of growth (t = 0 s), blebs are devoid of actin but CA-mDia1 is present. Scale bar represents 3 μm. (E) Kymograph of GFP-CA-mDia1 (green) and LifeAct-Ruby (red) localization taken along the dashed white line in (D). During bleb growth, no actin is visible under the bleb membrane (only green signal is visible; arrowhead), but an actin cortex reforms rapidly after bleb expansion stalls. (F) Localization of GFP-CA-mDia1 in the cortex of mitotic HeLa cells transiently expressing mCherry-β-actin. Scale bar represents 5 μm. (G) Half-times of fluorescence recovery after photobleaching for GFP-actin, GFP-CA-mDia1, GFP-Daam1, and GFP-GBD+FH3 domain at the cortex of M2 cells. Data are plotted as box-whisker plots, and the number of experiments is indicated on the graph. Whiskers indicate the maximum and minimum measurements. ^∗∗^p < 0.01. (H) Mean cortical fluorescence intensity in M2 cells stably expressing GFP-actin for nontransfected cells (wild-type) or cells transfected with mDia1 shRNA pre/posttreatment with CK666 or DMSO (controls). All data were normalized to the average fluorescence intensity prior to treatment. Data are derived from 30 cells for each experiment. Error bars indicate SEM. ^∗∗^p < 0.01. (I) Relative change in total cellular F-actin measured by phalloidin staining and flow cytometry for perturbations targeting mDia1 and the Arp2/3 complex. To minimize sample-to-sample variability, immunostaining and flow cytometry measurements were effected pairwise, with one population identified by carboxyfluorescein succinimidyl ester (CFSE) staining (see Supplemental Experimental Procedures). All perturbations led to significant changes in total cellular F-actin compared to controls. Errors bars indicate SEM. ^∗∗^p < 0.01. See also Figure S2.

We then investigated whether the formins Daam1 and mDia1 were bound to cortical F-actin. In apparent contradiction with our immunostaining data (Figures 1E and 1F), we have previously reported cytoplasmic localization for GFP-tagged mDia1 [16]. Therefore, we tested whether this was due to a high mDia1 unbound/bound ratio by imaging mDia1 dynamics in live cells using single-molecule imaging, a technique that enables visualization of the localization of proteins with high cytoplasmic background. mDia1-GFP speckles clearly localized to the cortex of blebs (Figure 2A, arrowheads) and mitotic HeLa cells (Figure 2B), remaining visible for several seconds before disappearing. To determine whether cortical speckles represented active mDia1 proteins, we imaged the localization of constitutively active mDia1 (CA-mDia1; Figure 2C). In contrast to full-length mDia1 that displays cytoplasmic localization [16], CA-mDia1 exclusively localized to the plasma membrane during all stages of the bleb life cycle in M2 cells (Figures 2D and 2E) and at the cortex of prometaphase HeLa cells (Figure 2F). Actin depolymerization did not alter CA-mDia1 localization (Figure S3A), indicating F-actin-independent recruitment. We concluded that mDia1 speckles primarily represented active proteins localized to the cortex or the cell membrane. To determine whether mDia1 and Daam1 bind to the actin cortex, we compared the half-time of fluorescence recovery after photobleaching of CA-mDia1 and Daam1 using actin as a positive control. The GTPase-formin homology 3 (FH3) domain of mDia1 (GBD+FH3), which lacks an actin-binding domain but localizes to the cell membrane (similar to the GTPase-binding domain of Daam1; Figures 2C, S3B, and S3C), was used as a negative control. We reasoned that binding to cortical F-actin should stabilize the cortical localization of formins and therefore slow down their fluorescence recovery compared to GBD+FH3. CA-mDia1 fluorescence recovered at a rate similar to F-actin, but ∼4-fold slower than GBD+FH3 and Daam1 (Figures 2G and S3D). Together, these results indicate that CA-mDia1 is strongly associated with the actin cortex but that Daam1 is not, suggesting that Daam1’s effect on bleb size in one cell line was indirect, perhaps by modulating cytoplasmic pressure or downregulating adhesion [21–24].

Overall, our independent screening and proteomic approaches identified the Arp2/3 complex and mDia1 as major contributors to the actin cortex, though contributions from Fhod1 and Daam1 cannot be entirely excluded, notably due to the incomplete mRNA depletion achieved in our experiments. We then focused on characterizing the specific functions of mDia1 and Arp2/3 in cortex generation, organization, and dynamics.

We first investigated what portion of the cortical F-actin was nucleated by each nucleator. CK666 treatment resulted in an ∼60% decrease in cortical GFP-actin fluorescence intensity compared to DMSO (Figures 1I and 2H). A similar effect was observed when mDia1-depleted cells were treated with CK666 compared to DMSO (Figure 2H), indicating that Arp2/3 still contributes to cortex formation when mDia1 is depleted. Consistent with this result, immunostaining revealed the presence of Arp2/3 at the cortex of mDia1 knockdown cells (Figure S3E). Conversely, mDia1 was present at the cortex of Arp2/3-depleted and CK666-treated cells (Figures S3F–S3I), suggesting that the localization of both nucleators was independent from one another. Flow cytometry measurements of the total F-actin fluorescence intensity in cell populations indicated that perturbation to mDia1 and Arp2/3 activity led to significant decreases in total F-actin fluorescence (Figure 2I). When both were perturbed simultaneously, total cellular F-actin decreased additively. Because both nucleators had strong effects on cortex stability and were localized to the cortex, we assumed that the decrease in total F-actin intensity observed using flow cytometry was entirely imputable to changes in cortical actin. Because cortical F-actin contributes roughly 50% of total cellular F-actin fluorescence measured in flow cytometry (n = 33 cells examined; Figure S3J), the ∼27.5% decrease observed in mDia1-depleted cells treated with CK666 suggests that, together, mDia1- and Arp2/3-mediated actin polymerization account for at least 55% of cortical F-actin in M2 cells (Figure 2I). The remainder may be due to residual activity of mDia1 or Arp2/3 as a result of incomplete depletion/inhibition or contributions from other less abundant nucleators.

**Figure 3:**
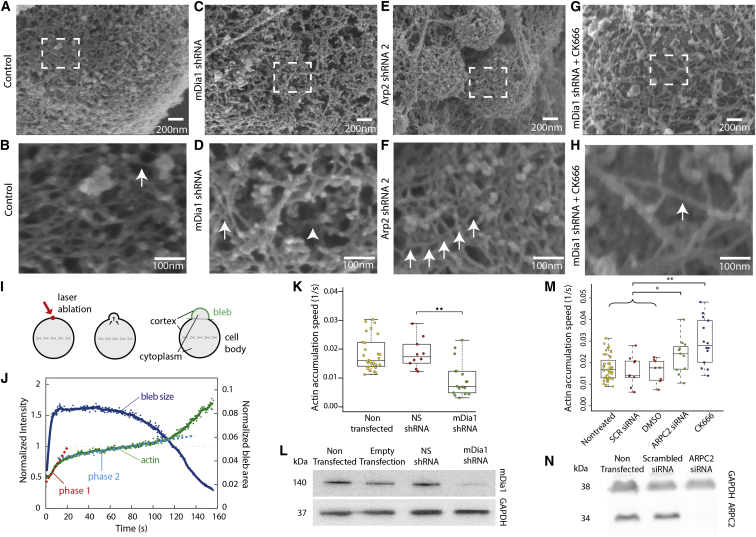
Effects of mDia1 and Arp2/3 Complex Depletion on Cortex Architecture and Assembly Dynamics (A–H) Representative scanning electron micrographs of the actin cortex at the surface of a bleb in detergent-extracted M2 cell untreated (A and B), stably expressing mDia1 shRNA (C and D), stably expressing Arp2 shRNA (E and F), or stably expressing mDia1 shRNA treated with the Arp2/3 complex inhibitor CK666 (G and H). (B), (D), (F), and (H) are magnifications of the boxed zones in (A), (C), (E), and (G), respectively. (B) The cortex is composed of a dense mesh of overlapping filaments primarily oriented tangential to the bleb surface. The actin filament density within the mesh appears approximately uniform. Individual filaments can clearly be distinguished (white arrow). (D) The cortex appears less uniform than in control cells with areas of high filament density (white arrow) alternating with large gaps devoid of filaments (arrowhead). (F) Filaments appear generally longer than in control cells and can be traced over several hundred nanometers (white arrows). A similar phenotype is observed for CK666 treatment. See Figure S3K. (H) The actin cortex is visibly less dense than in control cells. Only few actin filaments (white arrow) subsist with gaps of several hundred nanometers in between. (I) Single blebs are induced in metaphase HeLa cells by laser ablation of the cortex (red arrow). Automated image analysis is used to segment the cell into cytoplasm, cell body cortex, and bleb cortex and allows for precise measurement of the evolution of fluorescence intensity in these regions over time. (J) Representative actin regrowth curve as a function of time in a bleb induced by laser ablation in a metaphase HeLa cell expressing GFP-actin. The mean actin fluorescence intensity at the bleb cortex was normalized to the mean intensity in the cell body cortex (green). The evolution of bleb size with time is plotted in blue. Initial regrowth rates after ablation are linear with time (phase 1, red dashed line; phase 2, blue dashed line). t = 0 s, ablation onset. (K) Actin cortex accumulation rate in nontransfected control cells, cells transfected with NS, and cells transfected with mDia1 shRNA. Data are plotted as box-whisker plots and derived from nontreated control cells (n = 28), NS cells (n = 10), and mDia1 shRNA cells (n = 17). Whiskers indicate minimum and maximum actin accumulation rates. Data points are overlaid. ^∗∗^p < 0.01. (L) Immunoblot of HeLa cells transiently transfected with NS or mDia1 shRNA1 probed with anti-mDia1 and anti-GAPDH. (M) Actin cortex accumulation rate in nontreated control, DMSO-treated, CK666-treated, scrambled siRNA-treated, and ARPC2 siRNA-treated metaphase HeLa cells. Data are derived from nontreated control cells (n = 43), DMSO control cells (n = 9), CK666 cells (n = 14), scrambled siRNA cells (n = 9), and ARPC2 siRNA cells (n = 11). Whiskers indicate minimum and maximum actin accumulation rates. Data points are overlaid. ^∗^p < 0.05; ^∗∗^p < 0.01. (N) Immunoblot of HeLa cells transiently transfected with scrambled siRNA or ARPC2 siRNA probed with anti-ARPC2 and anti-GAPDH. See also Figure S3.

We next used scanning electron microscopy to examine the impact of nucleator depletion on cortex organization in blebs, because we reasoned that the effect would be most apparent in structures necessitating de novo cortical actin polymerization. Depletion of mDia1 led to extensive changes in cortical actin organization, with areas of high filament density (Figure 3C; white arrow in Figure 3D) alternating with large gaps (100–200 nm) devoid of actin filaments (arrowhead in Figure 3D), very different from the homogeneous mesh with a gap size of ∼30 nm observed in control conditions (Figures 3A and 3B). Arp2/3 complex inhibition and depletion both led to apparently longer filaments, easily distinguishable compared to control cortices (Figure 3E; arrows in Figure 3F; Figure S3K). When both nucleators were perturbed simultaneously, the actin cortex consisted of a loose array of poorly interconnected filaments with a mesh size > 100 nm (Figure 3G; arrow in Figure 3H).

Along with ultrastructural network organization, the dynamics of cortex assembly are a key determinant of the mechanical functions of the cortex in cell morphogenesis [7, 25, 26]. Therefore, we examined how mDia1 and the Arp2/3 complex participated in setting the rate of de novo cortical actin accumulation in mitotic HeLa cells, where bleb formation can be induced in a controlled manner by laser ablation of the cortex [27] (Figures 3I and 3J). De novo actin assembly in mDia1-depleted cells was 2-fold slower than in controls (Figures 3K and 3L). In contrast, perturbation of Arp2/3 activity by CK666 or ARPC2 small interfering RNA (siRNA) both led to an approximately 2-fold increase in actin assembly speed (Figures 3M and 3N). In control conditions, the actin assembly speed is the weighted average of the speeds of all nucleators. Thus, if a slower-than-average nucleator is depleted, assembly speed will increase, and conversely, if a faster-than-average nucleator is depleted, assembly speed will decrease. Hence, our results suggest that, consistent with rates of elongation measured for each nucleator in vitro [28, 29], mDia1-mediated assembly of cortical actin is substantially faster than average (Figure 3K), whereas that mediated by Arp2/3 is substantially slower (Figure 3M). Taken together, our results indicate that perturbing Arp2/3 and mDia1 leads to profound changes in actin cortex organization and dynamics (Figure 3).

**Figure 4:**
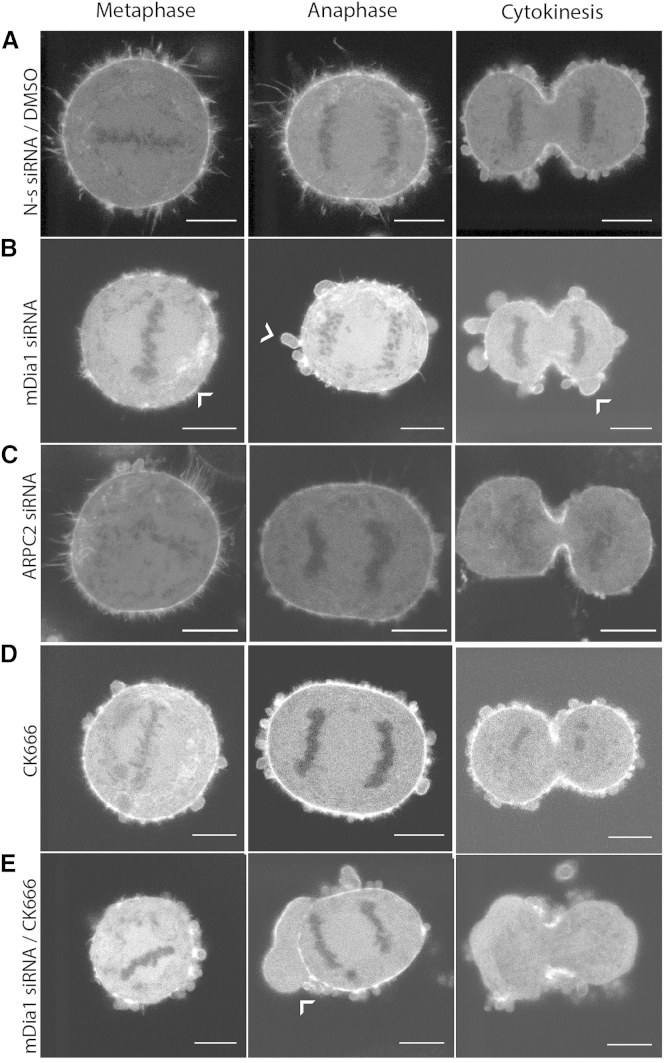
mDia1 Depletion Perturbs Actin Cortex Stability during Mitosis, and Its Effect Is Potentiated by Simultaneous Arp2/3 Complex Inhibition All panels are single confocal sections, and actin distribution during mitosis is visualized in HeLa cells stably expressing actin GFP. Scale bars represent 10 μm. (A) Actin distribution in representative cells at different stages of mitosis treated with nonsilencing siRNA and DMSO. (B) Actin distribution in representative cells at different stages of mitosis treated with mDia1 siRNA. The actin cortex appears less uniform (arrowhead; metaphase), and many large blebs form during anaphase and cytokinesis (arrowheads; anaphase and cytokinesis). See Figure S4D for immunoblot. (C) Actin distribution in representative cells at different stages of mitosis treated with ARPC2 siRNA. See Figure 3N for immunoblot. (D) Actin distribution in representative cells at different stages of mitosis treated with the Arp2/3 inhibitor CK666. (E) Actin distribution in representative cells at different stages of mitosis treated simultaneously with mDia1 siRNA and CK666. Blebs form at all stages of mitosis, and these are particularly large in anaphase (arrowhead). The polar cortex disappears completely in cytokinesis. See also Figure S4.

Finally, we asked how interfering with the activity of each nucleator affected cell morphogenesis, focusing on cell division, where cortex reorganization is known to drive dramatic shape changes. Although previous studies indicate a role for mDia1 and Arp2/3 in mitosis [30–32], their role in assembling the mitotic cortex remains unclear. We found that both mDia1 and Arp2/3 localized to the cell cortex at all stages of mitosis in HeLa cells (Figures S4A and S3B). CK666 treatment or ARPC2 depletion did not significantly affect progression through cytokinesis (Figures 3N and S4C). However, ARPC2 depletion gave rise to increased cell blebbing in interphase, consistent with previous studies [13, 33]. In contrast, mDia1 depletion significantly affected progression past mitosis, and the cortex appeared patchy (Figures 4B, S4C, and S4D). Simultaneous depletion of mDia1 and inhibition of Arp2/3 significantly accentuated these effects (Figure S4C). When examined by fluorescence microscopy, the cortex of cells depleted in ARPC2 or treated with CK666 remained homogeneous (Figures 4C and 4D), whereas cells in which we perturbed both mDia1 and Arp2/3 possessed significantly less cortical actin, their cortex appeared fragmented, and cells blebbed vigorously at all stages of mitosis (Figure 4E). In cytokinesis, the polar cortex disappeared nearly entirely (Figure 4E) and cell shape was very unstable, a phenotype reminiscent of that observed in M2 blebbing cells (Figure 1M). These experiments suggest that mDia1 and Arp2/3 play different roles in mitosis progression.

Our results demonstrate a crucial role for the formin mDia1 and the Arp2/3 complex in nucleating the submembranous F-actin cortex in M2 blebbing cells and HeLa cells. Although our experiments identify mDia1 and the Arp2/3 complex as major contributors to nucleation of the submembranous actin cortex, other studies suggest a possible role for different formins [8–10, 12, 14]. This, together with the greater tissue specificity of formins compared to the ubiquitous expression of the Arp2/3 complex, suggests that the importance of mDia1 may vary between cell types. Future studies will have to examine the generality of the role of mDia1 and explore potential complementary contributions from other nucleators, such as Fhod1, Daam1, and mDia2, to cortical actin generation. Whether mDia1 and Arp2/3 act independently from one another in the cortex remains unclear. Reports of synergistic action of pointed-end nucleators with barbed-end nucleators [34, 35] suggest that mDia1 could elongate new branches nucleated by Arp2/3, or that Arp2/3 could catalyze formation of branches from the side of filaments nucleated by mDia1. Some of our data support independent action. When we reduced the activity of each nucleator separately, M2 cells retained a well-defined cortex, but when we inhibited both simultaneously, most of the cortex disappeared (Figures 1G–1I and 1M). Furthermore, loss of F-actin due to simultaneous perturbation of mDia1 and Arp2/3 was comparable to the sum of the losses due to perturbation of each separately (Figure 2I). Finally, depletion of one nucleator did not affect localization of the other (Figures S3E–S3I). Together, these data suggest that the cortex is composed of two actin networks that grow concurrently but largely independently from one another. However, other observations point to synergistic action. During mitosis, mDia1 depletion resulted in an increase in cell division failures, and whereas Arp2/3 inhibition alone had no effect on cell division, it potentiated the effect of mDia1 depletion (Figures 4 and S4C). This suggests that mDia1 may act upstream of Arp2/3 in nucleating cortical actin. Future experiments will need to investigate the level of interdependency between mDia1- and Arp2/3-mediated actin assembly in the cortex, and whether this dependency is cell-cycle dependent.

Why generation of the cortex and other cellular F-actin structures, such as the leading edge of migrating cells [36], requires the combined action of two F-actin nucleators represents an intriguing question. Our ultrastructural examination suggests that the actin networks assembled by each nucleator (Figures 3A–3H) are not spatially separated as in the leading edge [36] but rather intertwined. Furthermore, perturbation of each nucleator had distinct effects on organization and kinetics (Figure 3), suggesting that each contributes differently to assemble a cortical F-actin network with the requisite mechanical properties. Nucleators likely affect cortical F-actin density, thickness, and organization, all of which influence the emerging physical properties of the cortical network [37]. Understanding how each nucleator contributes to cortical mechanics from the molecular level up will represent a challenge for the future.

## Supplemental Information

Document S1. Figures S1–S4, Table S1, and Supplemental Experimental Procedures

Movie S1. Representative Blebbing Cells Expressing Nonsilencing shRNA and shRNA Targeting mDia1 M2 blebbing cells stably expressing GFP-actin transfected with nonsilencing shRNA (left) or shRNA targeting mDia1 (right). Depletion of mDia1 (right) led to the formation of very large blebs compared to control cells (left), but the cells still retained a well-defined actin cortex. Transfected cells were identified based on the expression of a BFP marker present on the shRNA vector. Scale bar represents 5 μm. Total duration is 230 s.

Movie S2. Representative Blebbing Cells Expressing shRNA Targeting Arp2 and Arp3 M2 blebbing cells stably expressing LifeAct Ruby and stably transfected with shRNA targeting Arp2 (left) or shRNA targeting Arp3 (right). Depletion of Arp2 or Arp3 led to the formation of small blebs compared to control cells (Movie S1, left panel). Transfected cells were identified based on the expression of a GFP marker present on the shRNA vector. Scale bar represents 3 μm. Total duration is 600 s.

Movie S3. Representative Blebbing Cell Depleted in mDia1 prior to and during Treatment with Arp2/3 Complex Inhibitor M2 blebbing cell stably expressing GFP-actin transfected with shRNA targeting mDia1. Prior to treatment, the cell formed large blebs and retained a clear actin cortex. After treatment, the cell rapidly lost its shape and the majority of its cortical actin. The cell only retained a few discernible foci of cortical actin, and the nucleus was expelled from the cell body into a large bulge. Transfected cells were identified based on the expression of a BFP marker present on the shRNA vector. The timing of addition of CK666 is indicated on the movie. A 300 s pause occurred after addition of CK666 to allow for refocusing due to cell rounding in response to treatment. Images during this pause are not included in the movie. Scale bar represents 5 μm.

Document S2. Article plus Supplemental Information

## References

[1] Stewart M.P., Helenius J., Toyoda Y., Ramanathan S.P., Muller D.J., Hyman A.A. (2011). Hydrostatic pressure and the actomyosin cortex drive mitotic cell rounding. Nature.

[2] Kunda P., Pelling A.E., Liu T., Baum B. (2008). Moesin controls cortical rigidity, cell rounding, and spindle morphogenesis during mitosis. Curr. Biol..

[3] Bray D., White J.G. (1988). Cortical flow in animal cells. Science.

[4] Cramer L.P. (2010). Forming the cell rear first: breaking cell symmetry to trigger directed cell migration. Nat. Cell Biol..

[5] Levayer R., Lecuit T. (2012). Biomechanical regulation of contractility: spatial control and dynamics. Trends Cell Biol..

[6] Charras G., Paluch E. (2008). Blebs lead the way: how to migrate without lamellipodia. Nat. Rev. Mol. Cell Biol..

[7] Sedzinski J., Biro M., Oswald A., Tinevez J.Y., Salbreux G., Paluch E. (2011). Polar actomyosin contractility destabilizes the position of the cytokinetic furrow. Nature.

[8] Eisenmann K.M., Harris E.S., Kitchen S.M., Holman H.A., Higgs H.N., Alberts A.S. (2007). Dia-interacting protein modulates formin-mediated actin assembly at the cell cortex. Curr. Biol..

[9] Hannemann S., Madrid R., Stastna J., Kitzing T., Gasteier J., Schönichen A., Bouchet J., Jimenez A., Geyer M., Grosse R. (2008). The Diaphanous-related Formin FHOD1 associates with ROCK1 and promotes Src-dependent plasma membrane blebbing. J. Biol. Chem..

[10] Kitzing T.M., Wang Y., Pertz O., Copeland J.W., Grosse R. (2010). Formin-like 2 drives amoeboid invasive cell motility downstream of RhoC. Oncogene.

[11] Kitzing T.M., Sahadevan A.S., Brandt D.T., Knieling H., Hannemann S., Fackler O.T., Grosshans J., Grosse R. (2007). Positive feedback between Dia1, LARG, and RhoA regulates cell morphology and invasion. Genes Dev..

[12] Stastna J., Pan X., Wang H., Kollmannsperger A., Kutscheidt S., Lohmann V., Grosse R., Fackler O.T. (2012). Differing and isoform-specific roles for the formin DIAPH3 in plasma membrane blebbing and filopodia formation. Cell Res..

[13] Derivery E., Fink J., Martin D., Houdusse A., Piel M., Stradal T.E., Louvard D., Gautreau A. (2008). Free Brick1 is a trimeric precursor in the assembly of a functional wave complex. PLoS One.

[14] Wyse M.M., Lei J., Nestor-Kalinoski A.L., Eisenmann K.M. (2012). Dia-interacting protein (DIP) imposes migratory plasticity in mDia2-dependent tumor cells in three-dimensional matrices. PLoS One.

[15] Tominaga T., Sahai E., Chardin P., McCormick F., Courtneidge S.A., Alberts A.S. (2000). Diaphanous-related formins bridge Rho GTPase and Src tyrosine kinase signaling. Mol. Cell.

[16] Charras G.T., Hu C.K., Coughlin M., Mitchison T.J. (2006). Reassembly of contractile actin cortex in cell blebs. J. Cell Biol..

[17] Biro M., Romeo Y., Kroschwald S., Bovellan M., Boden A., Tcherkezian J., Roux P.P., Charras G., Paluch E.K. (2013). Cell cortex composition and homeostasis resolved by integrating proteomics and quantitative imaging. Cytoskeleton (Hoboken).

[18] Michelot A., Drubin D.G. (2011). Building distinct actin filament networks in a common cytoplasm. Curr. Biol..

[19] Poincloux R., Collin O., Lizárraga F., Romao M., Debray M., Piel M., Chavrier P. (2011). Contractility of the cell rear drives invasion of breast tumor cells in 3D Matrigel. Proc. Natl. Acad. Sci. USA.

[20] Nolen B.J., Tomasevic N., Russell A., Pierce D.W., Jia Z., McCormick C.D., Hartman J., Sakowicz R., Pollard T.D. (2009). Characterization of two classes of small molecule inhibitors of Arp2/3 complex. Nature.

[21] Luo W., Yu C.H., Lieu Z.Z., Allard J., Mogilner A., Sheetz M.P., Bershadsky A.D. (2013). Analysis of the local organization and dynamics of cellular actin networks. J. Cell Biol..

[22] Ang S.F., Zhao Z.S., Lim L., Manser E. (2010). DAAM1 is a formin required for centrosome re-orientation during cell migration. PLoS One.

[23] Jaiswal R., Breitsprecher D., Collins A., Corrêa I.R., Xu M.Q., Goode B.L. (2013). The formin Daam1 and fascin directly collaborate to promote filopodia formation. Curr. Biol..

[24] Saengsawang W., Taylor K.L., Lumbard D.C., Mitok K., Price A., Pietila L., Gomez T.M., Dent E.W. (2013). CIP4 coordinates with phospholipids and actin-associated proteins to localize to the protruding edge and produce actin ribs and veils. J. Cell Sci..

[25] Mayer M., Depken M., Bois J.S., Jülicher F., Grill S.W. (2010). Anisotropies in cortical tension reveal the physical basis of polarizing cortical flows. Nature.

[26] Robinson D.N., Spudich J.A. (2004). Mechanics and regulation of cytokinesis. Curr. Opin. Cell Biol..

[27] Tinevez J.Y., Schulze U., Salbreux G., Roensch J., Joanny J.F., Paluch E. (2009). Role of cortical tension in bleb growth. Proc. Natl. Acad. Sci. USA.

[28] Kovar D.R., Harris E.S., Mahaffy R., Higgs H.N., Pollard T.D. (2006). Control of the assembly of ATP- and ADP-actin by formins and profilin. Cell.

[29] Romero S., Le Clainche C., Didry D., Egile C., Pantaloni D., Carlier M.F. (2004). Formin is a processive motor that requires profilin to accelerate actin assembly and associated ATP hydrolysis. Cell.

[30] Mitsushima M., Aoki K., Ebisuya M., Matsumura S., Yamamoto T., Matsuda M., Toyoshima F., Nishida E. (2010). Revolving movement of a dynamic cluster of actin filaments during mitosis. J. Cell Biol..

[31] Kato T., Watanabe N., Morishima Y., Fujita A., Ishizaki T., Narumiya S. (2001). Localization of a mammalian homolog of diaphanous, mDia1, to the mitotic spindle in HeLa cells. J. Cell Sci..

[32] Dean S.O., Rogers S.L., Stuurman N., Vale R.D., Spudich J.A. (2005). Distinct pathways control recruitment and maintenance of myosin II at the cleavage furrow during cytokinesis. Proc. Natl. Acad. Sci. USA.

[33] Bergert M., Chandradoss S.D., Desai R.A., Paluch E. (2012). Cell mechanics control rapid transitions between blebs and lamellipodia during migration. Proc. Natl. Acad. Sci. USA.

[34] Okada K., Bartolini F., Deaconescu A.M., Moseley J.B., Dogic Z., Grigorieff N., Gundersen G.G., Goode B.L. (2010). Adenomatous polyposis coli protein nucleates actin assembly and synergizes with the formin mDia1. J. Cell Biol..

[35] Quinlan M.E., Hilgert S., Bedrossian A., Mullins R.D., Kerkhoff E. (2007). Regulatory interactions between two actin nucleators, Spire and Cappuccino. J. Cell Biol..

[36] Ridley A.J. (2011). Life at the leading edge. Cell.

[37] Salbreux G., Charras G., Paluch E. (2012). Actin cortex mechanics and cellular morphogenesis. Trends Cell Biol..

