# Supplementary material for: Cellular Control of Cortical Actin Nucleation
Source: Curr Biol. 2014 Jul 21;24(14):1628–35. doi: 10.1016/j.cub.2014.05.069 (PMC4110400; doi:10.1016/j.cub.2014.05.069)
Supplement: Document S1. Figures S1–S4, Table S1, and Supplemental Experimental Procedures [file mmc1.pdf]

Current Biology, Volume *24*

Supplemental Information

Cellular Control
of Cortical Actin Nucleation

Miia Bovellan, Yves Romeo, Maté Biro, Annett Boden, Priyamvada Chugh, Amina Yonis, Malti Vaghela, Marco Fritzsche, Dale Moulding, Richard Thorogate, Antoine Jégou, Adrian J. Thrasher, Guillaume Romet-Lemonne, Philippe P. Roux, Ewa K. Paluch, and Guillaume Charras

Supplemental Data

Supplemental Figures and Legends

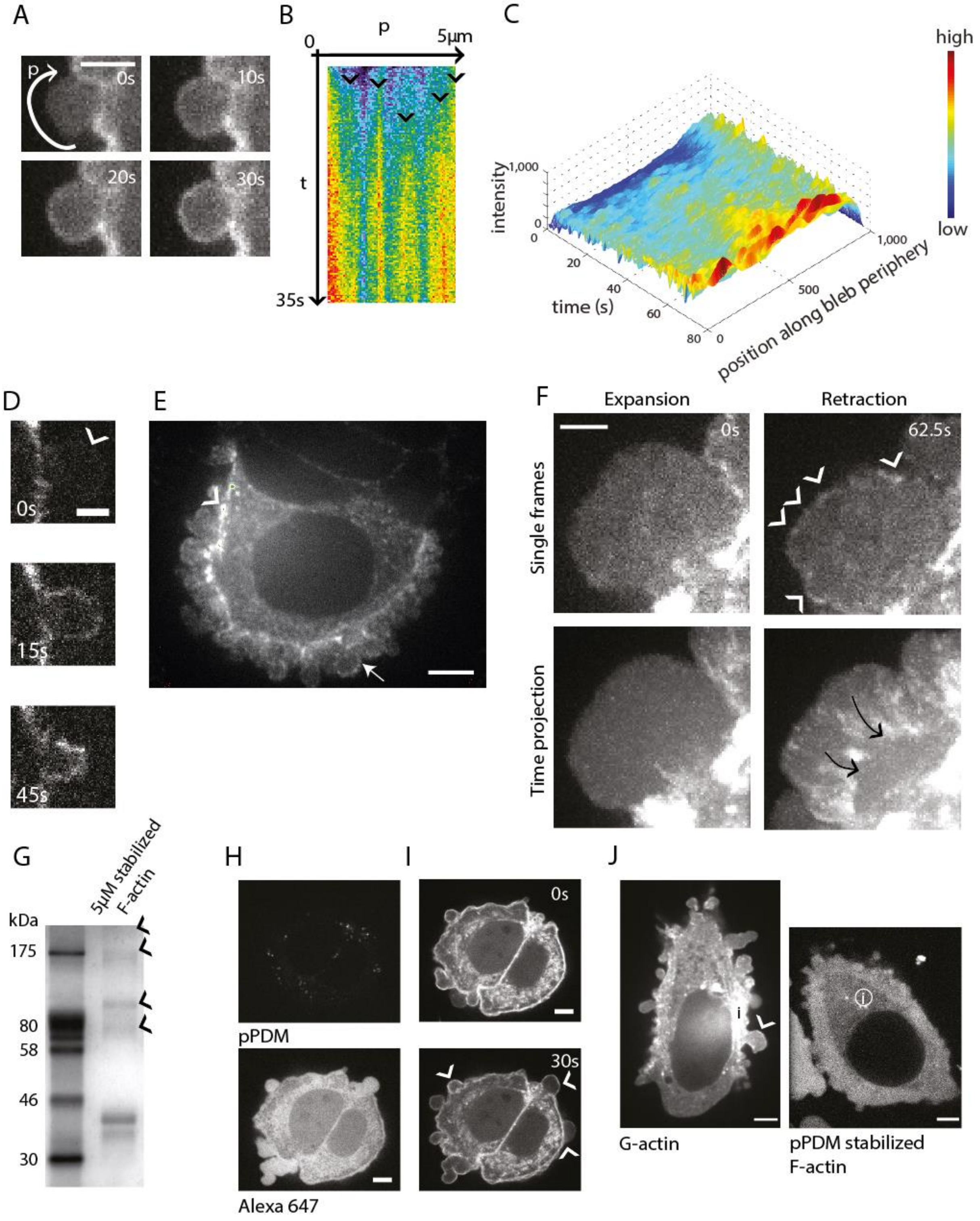

Figure S1 related to Figure 1| Cortex regrowth at the bleb membrane does not occur by actin seed elongation.

A-C: The actin cortex does not invade the bleb from pre-existing cortex at the bleb base. If lateral cortical invasion significantly contributed to actin regrowth, we would expect to observe preferential regrowth of cortex from the bleb base in time-lapse movies of F-actin

localisation. If this model were correct, we should first observe regrowth from membrane sites situated closest to the bleb neck, while regrowth would occur last in the centre of the bleb at the point furthest from the bleb neck. In experimentally acquired kymographs of the fluorescence intensity of the F-actin reporter LifeAct along the bleb membrane (**A**), we did not observe this. Rather, F-actin accumulated from seemingly random locations under the bleb membrane in both M2 and HeLa cells (**B**-**C**).

A. Actin cortex regrowth in a bleb in an M2 cell stably expressing LifeAct Ruby. Timings are given in sec. Scale bar 3μm. **B.** Kymograph of LifeAct-Ruby fluorescence intensity under the bleb membrane for the cell shown in **A**. Recording was started at the end of expansion and stopped when a clear visible cortex was detected. Time is shown on the vertical axis (indicated as *t*) and the position along the bleb perimeter is shown on the horizontal axis (indicated as *p* on **A**). Hot colours represent high fluorescence intensities and cold colours low intensities. The locations where cortical regrowth is first initiated after expansion are indicated with arrowheads. Similar results were seen in all blebs studied (n= 20 blebs, n=8 cells). **C.** Representative kymograph of LifeAct-mCherry fluorescence intensity under the membrane of a bleb induced by laser ablation in a mitotic HeLa cell. Hot colours represent high fluorescence intensities, denoting an actin-rich cortex, and cold colours low intensities. Fluorescence intensities are also shown on the vertical z-axis, time from the onset of bleb formation is shown on the y-axis and position along the bleb periphery is shown on the x-axis. Similar results were seen in all blebs studied (n= 20 cells).

D-F: The cortex does not regrow from small actin fragments remaining under the bleb membrane.

To investigate the presence of bound-seeds under the bleb membrane, we microinjected phalloidin functionalised quantum dots (Qdots) into cells. First, we verified that microinjection of small amounts of rhodamine-tagged phalloidin did not perturb the bleb life cycle (**D**). In newly formed blebs, no phalloidin localization could be observed (**D**, t=0s) but after growth stopped, phalloidin localized to the bleb membrane indicating the presence of F-actin (**D**, t=15s). This transition in localization mirrored in all points what we had previously observed with GFP-Actin [S1]. Phalloidin localization persisted during bleb retraction (**D**, t=45s). We then turned to phalloidin functionalized Qdots, which could help detecting small patches of F-actin because single Qdots are easily detectable via standard fluorescence microscopy techniques. Phalloidin-Qdots microinjected into cells were present in two subpopulations: a mobile subpopulation and an immobile subpopulation. The immobile subpopulation localized to regions enriched in F-actin (arrowhead, **E**), consistent with other experiments utilizing phalloidin functionalized Qdots [S2]; such localization is not observed in cells microinjected with PEG-passivated Qdots [S3, 4]. The mobile subpopulation diffused throughout the cytoplasm. Qdots penetrated into the bleb cytosol from the onset of blebbing and accumulated under the membrane before retraction (**D-F**). We reasoned that if F-actin seeds remain bound to the cell membrane during bleb growth, we should observe Qdots following the bleb membrane and, in time-projections, their trajectories should appear as streaks of fluorescence. In our experiments, bound Qdots were never detected during expansion (n=0 out of 75 blebs examined, panel **F**, left column); whereas, during retraction, many streaks were observed – consistent with the presence of an actin cortex during retraction (n=75 out of 75 blebs examined, panel **F**, right column lower row, arrows). This indicates that no F-actin seeds stay bound to the bleb membrane during expansion.

D. F-actin localisation during expansion (t=0s) and retraction (t=15-45s) of a bleb in an M2 cell visualised with rhodamine-phalloidin microinjected into the cell. Timings are given in sec.

Scale bar 3μm. **E.** Representative Quantum dot (Qdot)–phalloidin localisation in an M2 cell. Qdots accumulate at the cell cortex (arrowhead) and under the membrane of retracting blebs (arrow). Scale bar 5 μm. **F.** Representative Qdot–phalloidin localisation in an M2 cell bleb during expansion and retraction. Upper row images show single frames of a time-lapse movie at the end of expansion and during retraction. Bound Qdots are indicated by arrowheads. Lower row images show a projection of all the time frames during the expansion and retraction phases of a bleb. In time-projections, the trajectories of Qdots bound to the membrane appear as streaks (trajectories delineated by arrows). Similar results were seen in all blebs studied (n= 75 blebs from 15 cells). Scale bar 3μm.

G-J: Small actin filaments are not captured by membrane-actin linker proteins at the cell membrane.

Finally, we envisaged a mechanism in which small actin filaments diffuse into the bleb and are captured by membrane-actin linker proteins such as ezrin. To examine this hypothesis, we microinjected exogenous F-actin seeds in blebbing cells and asked whether they would become recruited to the bleb membrane. We confirmed experiments showing that exogenous F-actin seeds do not induce ectopic actin polymerization in cells. F-actin seeds were produced by incubating Alexa-488 labeled F-actin with p-NN'-phenylenebismaleimide (pPDM, **G**) to create covalently crosslinked protomers that act as caps at either end of the seed to block or slow depolymerization [S5, 6]. In vitro work has shown that these pPDM-crosslinked actin filaments can be elongated [S7] and actin-associated proteins can bind to them [S8, 9]. pPDM alone was not autofluorescent (**H**) and its microinjection did not interfere with blebbing (**I**). When we microinjected stabilised fluorescent actin seeds into cells, they remained diffuse and never concentrated at the cell cortex (n=44 out of 44 cells examined, **J** - right); in contrast, microinjected Alexa-488 G-actin incorporated into the cortex of cells and blebs (n=12 out of 12 cells examined, **J** – left, arrowhead), indicating that microinjected Alexa-488 G-actin could be readily incorporated into the bleb cortex [S10] but that pre-formed seeds could not. The lack of elongation of microinjected prepolymerised seeds is consistent with other reports[S11]. We concluded that pre-formed F-actin seeds did not contribute to cortex regrowth at the bleb membrane.

G. SDS-PAGE gel of pPDM stabilised actin filaments (indicated as 5μM stabilized F-actin) used for the microinjections in panel **J**. Covalently crosslinked actin protomers denoting dimers, trimers, tetramers, and pentamers are indicated by arrowheads. **H.** Fluorescence image of M2 cells stably expressing LifeAct-Ruby microinjected with 0.3mM pPDM together with Alexa-647 labelled dextran, to confirm micro-injection. Top: fluorescence image after excitation with 488nm light, collecting emission at 525nm, indicating that pPDM alone has no autofluorescence under these imaging conditions (control for **J**). Bottom: fluorescence image after excitation with 647nm light, collecting emission at 670nm, indicating that the cell has been microinjected. Scale bar 5μm. **I.** Same cells as in **G** showing the actin cytoskeleton revealed by the LifeAct-Ruby reporter exciting at 568nm and collecting emission at 610nm. New blebs continue to form (arrowheads) after micro-injection of pure pPDM. Scale bar 5μm. **J.** Left: Localisation of microinjected fluorescently labelled monomeric actin (Alexa-488 labeled G-actin, 10μM microinjection solution). G-actin localises to the cortex in the cell body and in blebs (arrowhead). Right: Localisation of microinjected stabilised actin seeds (microinjection solution: 5μM solution of pPDM stabilized Alexa-488 labelled actin filaments). No recruitment to the cortex was observed. Images are single frames of a time-lapse movie acquired using confocal microscopy. The site of injection is marked with the letter i. Scale bars 5μm.

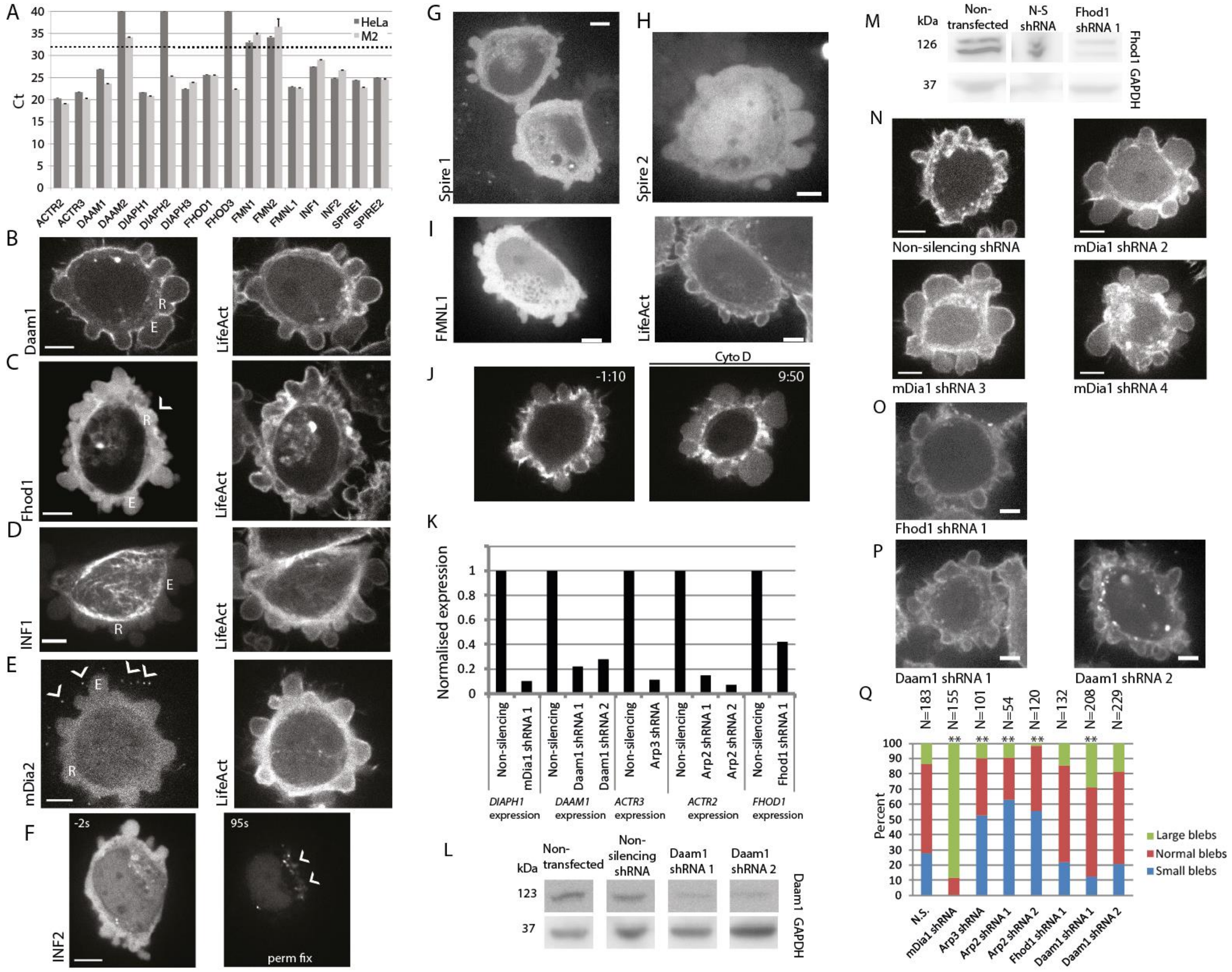

Figure S2 related to Figure 2| Expression and localization of F-actin nucleators and cellular phenotypes upon nucleator depletion.

A. mRNA abundance for actin nucleators in M2 and HeLa cells determined by qPCR and normalised to GAPDH mRNA abundance. The graph shows the number of cycles (Ct) needed to obtain detectable transcripts. Genes were not considered expressed above 32 cycles (dashed line). Data is averaged over 3 independent qPCRs. **(B-I)** Panels are single optical sections acquired by live confocal microscopy. 'E' indicates an expanding bleb and 'R' a retracting bleb. **B.** Localisation of GFP-Daam1 in an M2 cell stably expressing LifeAct-Ruby. Daam1 is present at the bleb membrane at all time points. Scale bar 5µm. **C.** Localisation of GFP-Fhod1 in an M2 cell stably expressing LifeAct-Ruby. An arrowhead indicates the enrichment of Fhod1 in retracting blebs. Scale bar 5µm. **D.** Localisation of GFP-INF1 in an M2 cell stably expressing LifeAct-Ruby. Scale bar 5µm. **E.** Localisation of GFP-mDia2 (*DIAPH3* gene) in an M2 cell stably expressing LifeAct-Ruby. Arrowheads indicate the enrichment of mDia2 at the tips of filopodia. Scale bar 5µm. **F.** Localisation of GFP-INF2 in an M2 cell revealed by simultaneous permeabilization and fixation to decrease cytoplasmic background. A solution of 0.25% glutaraldehyde and 0.5% Triton X-100 was added at time point 0s. Arrowheads indicate the enrichment of INF2 at the endoplasmic reticulum, consistent with [S12]. Scale bar 5µm. **G.** Localisation of GFP-Spire1 in an M2 cell. Scale bar 3µm. **H.** Localisation of mCherry-Spire2

in an M2 cell. Scale bar 3µm. **I.** Localisation of GFP-FMNL1 in an M2 cell stably expressing LifeAct-Ruby. Scale bar 3µm. **J.** Cytochalasin treatment of M2 blebbing cells stably expressing Actin-GFP. 40nM Cytochalasin D was added at time point 0:00. After cytochalasin addition, cells formed noticeably larger blebs that still reformed an actin cortex and retracted. Time in min:s. Scale bar 5µm. **K.** mRNA abundance in M2 cells stably expressing shRNA constructs targeting mDia1, Arp2, Arp3, Daam1, and Fhod1. The graph shows mRNA expression levels relative to control non-silencing shRNA construct (RQ). Abundance was normalised to GAPDH mRNA levels. Differences were considered significant if relative mRNA abundance (RQ) was reduced by at least 40%. Data is averaged over two independent experiments. **L.** Immunoblot of M2 cells stably transfected with non-silencing shRNA, Daam1 shRNA1, or Daam1 shRNA2 probed with anti-Daam1 and anti-GAPDH. **M.** Immunoblot of M2 cells stably transfected with non-silencing shRNA or Fhod1 shRNA1 probed with anti-Fhod1 and anti-GAPDH. **N.** Representative phenotypes for cells transiently transfected with non-silencing shRNA, mDia1 shRNA2, mDia1 shRNA3, and mDia1 shRNA4 in M2 blebbing melanoma cells expressing Lifeact-Ruby. A representative phenotype for mDia1 shRNA1 is shown on **Fig. 1G** and in **Movie S1**. Cells expressing shRNA targeting mDia1 had significantly larger blebs than cells expressing non-silencing shRNA. Scale bar 5µm. **O.** Representative phenotype for cells stably expressing Fhod1 shRNA1 in M2 blebbing melanoma cells expressing Lifeact-Ruby. Scale bar 3µm. **P.** Representative phenotypes for cells stably expressing Daam1 shRNA1, and Daam1 shRNA2 in M2 blebbing melanoma cells expressing Lifeact-Ruby. Scale bar 3µm. **Q.** Distribution of bleb sizes in M2 melanoma cells stably expressing Non-Silencing shRNA, mDia1 shRNA1, Arp2 shRNA1, Arp2 shRNA2, Arp3 shRNA1, Fhod1 shRNA1, Daam1 shRNA1, and Daam1 shRNA2. The number of cells examined in each experiment is indicated above each column. When compared to cells stably expressing Non-Silencing shRNA, cells expressing mDia1 shRNA1, Arp3 shRNA1, Arp2 shRNA1, Arp2 shRNA2, and Daam1 shRNA1 showed significantly different bleb distributions (**, $p<0.01$). In contrast, cells expressing Daam1 shRNA2 and Fhod1 shRNA1 did not display a significantly different bleb distributions compared to cells expressing Non-Silencing shRNA ($p=0.03$ and $p=0.54$, respectively). Statistical analysis and raw data are reported in **Table S1**.

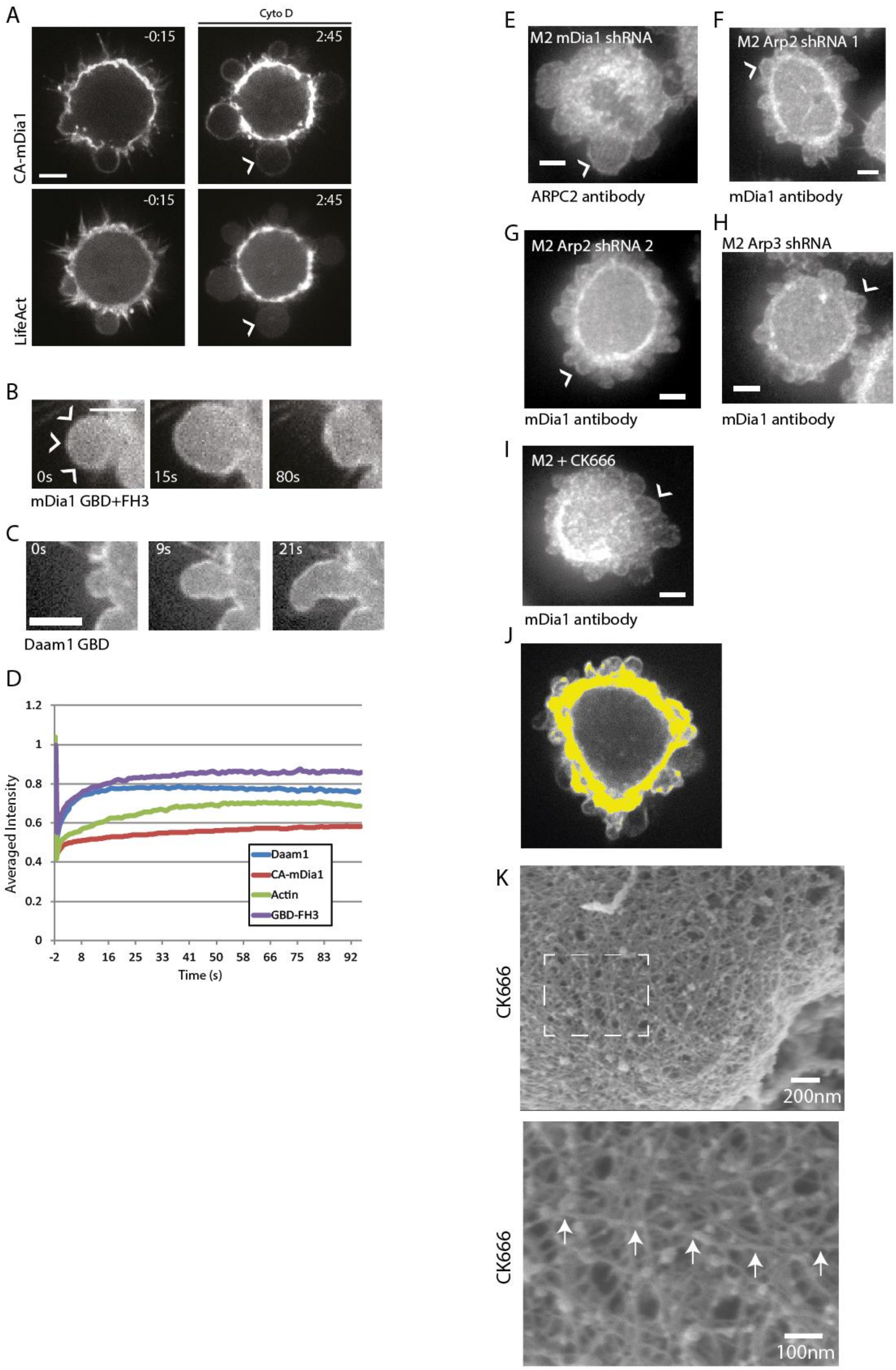

Figure S3 related to Figure 3 | mDia1 recruitment to the cortex, nucleator localization in cells expressing shRNA targeting mDia1 or the Arp2/3 complex, and organization of the actin cortex in cells treated with CK666.

A. Localisation of GFP-CA-mDia1 in M2 blebbing cells stably expressing LifeAct-Ruby upon Cytochalasin treatment. 5µM Cytochalasin D was added at time point 0:00. Blebs formed after

cytochalasin addition (arrowhead) did not reform an actin cortex and did not retract; CA-mDia1 still localized to their membrane, indicating that its recruitment did not depend on the presence of F-actin. Time in min:s. Scale bar 5μm. **B.** Localisation of the GBD+FH3 domain of mDia1 in an M2 cell bleb during different phases of the bleb life cycle (expansion: t=0s, end of growth: t=15s, retraction: t=80s). Scale bar 3μm. **C.** Localisation of the GTPase binding domain of Daam1 (Daam1 GDB) in an M2 cell bleb during different phases of the bleb life cycle (expansion: t=0s, end of growth: t=45s). Scale bar 3μm. **D.** Average fluorescence recovery curves after photobleaching for GFP-actin, GFP-Daam1, GFP-CA-mDia1 and GFP-GBD+FH3 at the cortex of M2 cells. Graphs are averaged over 25, 33, 14, and 15 cells respectively. (**E-I**) Single plane confocal images. Scale bars 3μm. **E.** Immunofluorescence image of an M2 blebbing cell stably expressing mDia1 shRNA1 stained with anti-ARPC2. **F.** Immunofluorescence image of an M2 blebbing cell stably expressing Arp2 shRNA1 stained with anti-mDia1. **G.** Immunofluorescence image of an M2 blebbing cell stably expressing Arp2 shRNA2 stained with anti-mDia1. **H.** Immunofluorescence image of an M2 blebbing cell stably expressing Arp3 shRNA stained with anti-mDia1. **I.** Immunofluorescence image of an M2 blebbing cell treated with 100μM CK666 stained with anti-mDia1. **J.** Representative M2 cell stained with Rhodamine Phalloidin. Fluorescence was segmented into cortical and cytoplasmic based on intensity. Cortical fluorescence is presented as a yellow overlay and accounts for approximately 50% of total cellular fluorescence. **K**. Top panel: Representative scanning electron micrograph of the actin cortex in a detergent extracted cell treated with the Arp2/3 complex inhibitor CK666. Bottom panel: Magnification of the boxed zone in the top panel. Filaments appear generally longer than in control cells and can be traced over several hundred nanometres (white arrows).

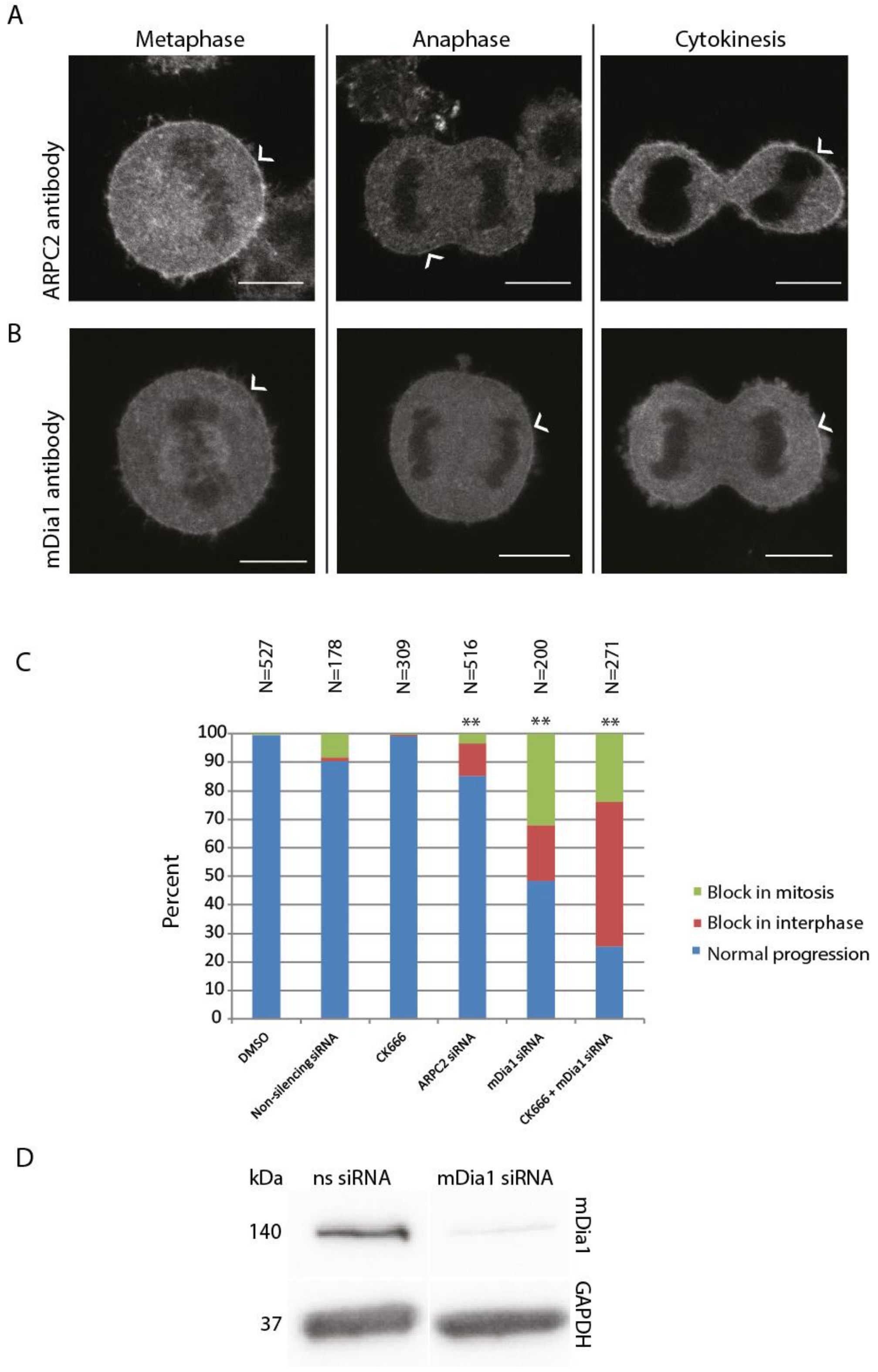

Figure S4 related to Figure 4 | mDia1 and the Arp2/3 complex localisation and function during mitosis progression.

A. Representative immunofluorescence images of HeLa cells at different stages of mitosis stained with anti-ARPC2. Arrows point out cortical localisation. Scale bars 10µm. **B.** Representative immunofluorescence images of HeLa cells at different stages of mitosis stained with anti-mDia1. Arrows point out cortical localisation. Scale bars 10µm. **C.** HeLa cells categorized by their progression or failure to progress through the cell cycle for different treatments. The number of cells examined in each experiment is indicated above each column.

When compared to cells transfected with Non-Silencing siRNA, cells transfected with mDia1 siRNA, ARPC2 siRNa, and mDia1 siRNA+CK666 showed significantly reduced rates of successful cell cycle progression (**, $p<0.01$). In contrast, cells treated with CK666 did not display a significantly different rate of successful cell cycle progression compared to cells treated with DMSO ($p=0.37$). **D.** Immunoblot of HeLa cells transfected with non-silencing siRNA or mDia1 siRNA probed with anti-mDia1 and anti-GAPDH.

Supplemental tables

Table S1: Raw data and statistical analysis of the phenotypes in the shRNA screen

**PHENOTYPE**	**Observed (number of cells in each category)**	**Expected number of cells in each category based on control**	**P – VALUE (CHITEST)**
	**CONTROL (non-silencing shRNA)**		
**Small blebs**	51		
**Normal blebs**	107		
**Large blebs**	25		
	**DAAM1 shRNA1**		
**Small blebs**	26	58	<0.001
**Normal blebs**	122	122	
**Large blebs**	60	28	
	**DAAM1 shRNA2**		0.03
**Small blebs**	47	64	
**Normal blebs**	139	134	
**Large blebs**	43	31	
	**Fhod1 shRNA1**		
**Small blebs**	29	37	0.51
**Normal blebs**	84	77	
**Large blebs**	19	18	
	**DIAPH1 shRNA**		
**Small blebs**	0	43	<0.001
**Normal blebs**	18	91	
**Large blebs**	137	21	
	**ARP2 shRNA1**		
**Small blebs**	34	15	<0.001
**Normal blebs**	15	32	
**Large blebs**	5	7	
	**ARP2 shRNA2**		
**Small blebs**	67	33	<0.001
**Normal blebs**	51	70	
**Large blebs**	2	16	
	**ARP3 shRNA1**		
**Small blebs**	53	28	<0.001
**Normal blebs**	38	59	
**Large blebs**	10	14	

Cells stably expressing the shRNA construct of interest were categorized as normal, displaying large blebs, and displaying small blebs. For each targeting shRNA construct, observed distributions were compared to expected numbers based on the categorization observed in non-silencing shRNA control using a chi-square test. Differences were considered significant for $p<0.01$.

Supplemental Experimental Procedures

Cell culture and generation of cell lines

M2 melanoma cells were a kind gift of Prof Tom Stossel (Harvard Medical School, Boston, USA) and were originally described in [S13]. Cell lines were cultured in MEM with Earle's salts and L-Glutamine (PAA) with penicillin/streptomycin, and 10% 80:20 mix of newborn calf serum/fetal bovine serum. For generation of M2 cells stably expressing the F-actin reporter protein LifeAct-Ruby (a gift from R. Wedlich-Söldner, MPI-Biochemistry, Martinsried, Germany), we excised LifeAct-Ruby and inserted it into the pLNCX2 retroviral vector (Takara-Clontech). For generation of cells expressing GFP-actin, we excised actin from EGFP-actin (Takara-Clontech) and inserted it into pRetroQ-AcGFP-C1 (Takara-Clontech). These plasmids were transfected into 293-GPG cells for packaging (a kind gift from Daniel Ory, Washington University, [S14]). Retroviral supernatants were then used to infect wild type M2 cells, the cells were selected in the presence of 1mg/ml G418 (LifeAct-Ruby, Merck Biosciences UK, Nottingham, UK) or 250ng/ml puromycin (GFP-actin) for 2 weeks and subcloned to obtain a monoclonal cell line. To obtain stable protein knock-down cell lines, M2 cells stably expressing LifeAct-Ruby or GFP-Actin were transfected, according to the protocol described below, with the appropriate shRNA plasmids (see plasmid construction and transfection) that had been linearised by digestion with *Ssp*I. The cells were then selected with 250ng/ml puromycin for two weeks, and subcloned.

The stable HeLa GFP-Actin line was a gift from F. Buchholz (MPI-CBG, Dresden, Germany) and the HeLa LifeAct-Ruby line was generated similarly to M2 LifeAct-Ruby. HeLa cells stably expressing LifeAct-Ruby or GFP-actin were cultured in DMEM (PAA or Gibco) with penicillin/ streptomycin, L-Glutamine, 10% fetal bovine serum and 750µg/ml G418. HeLa cells were arrested in metaphase with 100nM Nocodazole (Merck Biosciences) for 16h for the localisation studies. For metaphase arrest in laser ablation studies, cells were treated for at least 1 hour with 10µM MG132 (Sigma, St Louis, MO, USA).

All imaging was done in Leibovitz L-15 media (PAA) supplemented with 10% fetal calf serum or in phenol-red-free DMEM (Gibco) supplemented with 10% fetal bovine serum, L-Glutamine and penicillin/streptomycin.

All cell lines were cultured at 37°C with 5% CO_2. All lines were routinely screened for the presence of mycoplasma by DAPI staining.

Plasmid construction and transfection

A DNA fragment corresponding to the full-length human *DIAPH1* cDNA (GeneID 1729) was amplified from an I.M.A.G.E. full length cDNA clone, ID:40125808 (Source BioScience, Nottingham, UK) by PCR. Constitutively active (CA) mDia1 (aa 1-1141), GBD (aa 1-260), and GBD+FH3 (aa 1-464) were obtained by PCR from the full length human *DIAPH1*. The oligonucleotides used in the amplification of full-length human mDia1, CA-mDia1, GBD, and GBD+FH3 created *Xho*I and *Kpn*I sites at the 5′ and 3′ ends of the PCR fragment. Fragments were digested and ligated into the pEGFP-C1 (Clontech) vector.

Gene	Gene ID	I.M.A.G.E. ID	Restriction sites	Other source
ARPC1	10095	2967684	*EcoR*I *BamH*I	
DAAM1	23002		*Nco*I *Not*I	Mammalian Gene Collection
DIAPH3	81624	4830888	*EcoR*I *Apa*I	
FMNL1	752	4343469	*Hind*III *BamH*I	
INF1	229474	8860651	*Sal*I *Kpn*I	
INF2	64423	4053416	*Hind*III *BamH*I	

In the table, the name of the gene cloned, the geneID, I.M.A.G.E. ID (Source BioScience, Nottingham, UK), restriction sites used, and the possible other source of plasmid is described.

Full length *DAAM1*, *DIAPH3, FMNL1, INF1, INF2*, and *ARPC1* were obtained from the Mammalian Gene collection or the I.M.A.G.E. library and cloned into EGFP-C1 or EGFP-N1 vectors using restrictions sites inserted by PCR. Spire1-GFP and Spire2-mCherry were kind gifts of Dr Isabelle Tardieux (Institut Cochin, Paris, France, [S15]), and FHOD1-GFP was a kind gift from Dr Joyce Fingeroth (Harvard Medical school, [S16]). Speckling constructs were generated by introducing the gene of interest into a pEGFP vector harboring a truncated CMV promoter (a kind gift from Naoki Watanabe, [S17]). All gene products were verified by sequencing. For measurement of the cellular F-actin content, CA-mDia1 was also subcloned into pEBFP2-C1 (EBFP2 was obtained from Addgene, plasmid 14893, [S18]).

A typical transfection mixture for one well of a 6-well plate contained 1000µl Optimem (Invitrogen), 2.5µl Lipofectamine 1000 (Invitrogen), and 1µg of plasmid DNA.

Isolation of separated blebs

For bleb isolation for proteomics, we followed the protocol detailed in [S19]. Briefly, cells grown to confluence in T75 tissue culture flasks were exposed to 2 ml of medium containing 750 nM of the actin-depolymerizing drug Latrunculin B. Latrunculin-induced blebs were then separated by agitation at top speed on a rotary shaker for 15 min, the supernatant was collected, and pelleted at 13,000 g for 5 min. Pelleted blebs were then resuspended in 100 µl of Leibovitz L15. Several flasks were aggregated at this stage and the suspension was layered onto a density gradient with steps containing 5%, 12.5%, and 16% Ficoll in L15. The tubes were centrifuged at 100,000g for 40 minutes to separate blebs from contaminating whole cells. After centrifugation, separated blebs were collected from the 5%-12.5% interface. The collected fraction was then homogenized and washed in L15. Separated blebs were then resuspended in a low calcium (500nM) intracellular buffer (5 mM NaCl, 140 mM K-Glutamate, 7 mM $MgCl_2$, 6.7 mM $CaCl_2$, 10.2 mM K-EGTA, 20 mM K-Hepes, 10 mg/ml BSA, pH 7.2) containing an exogenous ATP regeneration system based on creatine kinase hydrolysis of creatine phosphate (Energy mix: 1 mM ATP, 1mM UTP, 1mM $MgCl_2$, 10mM creatine phosphate (Merck), 1 mg/ml creatine phosphokinase (Merck)). To allow for penetration of ATP into the separated blebs, small pores were generated in separated bleb membranes by addition of 50 µg/ml Staphylococcus Aureus α-toxin (Hemolysin, Sigma) and incubated at room temperature for 30 min.

Mass Spectrometry and Data Analysis

To generate purified cortical fractions, blebs isolated from 1×10^8 M2 cells were lysed in mild detergent (10 mM K_3PO_4, 1 mM EDTA, 5 mM EGTA, 10 mM $MgCl_2$, 50 mM β-glycerophosphate, 0.5% Nonidet P-40, 0.1% Brij 35, 0.1% deoxycholic acid, 1 mM sodium orthovanadate (Na_3VO_4), and 1 mM phenylmethylsulfonyl fluoride with a complete protease inhibitor cocktail tablet (Roche)). Bleb cortices were isolated by centrifugation at 14,000 × g

to remove cytoplasmic and membranous proteins. Proteins were then denatured in 2× reducing sample buffer and subjected to SDS-PAGE. For MS analysis, coomassie-stained gel bands were excised at indicated molecular weights and subjected to in-gel trypsin digestion, as described previously in Ref.[S20]. The resulting peptides were extracted and subjected to capillary LC-MS/MS using a high resolution hybrid mass spectrometer LTQ-orbitrap XL (Thermo Fisher Scientific). Experiments were performed in triplicate. Database searches were performed against a non-redundant International Protein Index (IPI) human database (version 3.23; containing 51 536 sequences and 24 497 860 residues) using Mascot (version 2.1; http://www.matrixscience.com). For data analysis, all proteins of the same family were grouped and isoforms of the same protein were considered as one. Two criteria were applied for considering a protein to be a reproducibly identified component of bleb cortex: 1) the presence of three or more sequence counts and a false positive protein identification rate of less than 1; 2) detection of the protein (using criteria 1) in at least two out of three replicate runs. To estimate the abundance of proteins in bleb cortex we rely on Protein Abundance Index (PAI) [S21] in a modified form. PAI was calculated based on spectral count as follows: PAI = spectral count/MW, where MW corresponds to the protein molecular weight, which was used to adjust for differences between proteins in the number of observable peptides. The identified proteins were then curated for actin nucleators and actin nucleation promotion factors.

shRNA transfection, constructs and targeted screen analysis
shRNAmir constructs in pGIPZ vectors targeting Daam1, mDia1, ACTR2, ACTR3, and Fhod1, as well as non-silencing shRNA, were obtained from Open Biosystems. Transfected cells can be detected by expression of a GFP reporter contained in the pGIPZ vector (Open Biosystems). In some experiments, the GFP in the pGIPZ shRNAmir constructs was replaced with EBFP2 (Addgene plasmid 14893, [S18]) by using *SnaB*I and *BsrG*I sites. The shRNAmir construct gene accessions, oligo Ids, target sequences and transfection protocol are given in the next table.

Transient transfection of shRNA: For shRNA screening, a typical transfection mixture for one well of a 6-well plate contained 1000μl Optimem (Invitrogen), 2.5μl Lipofectamine 2000 (Invitrogen), and 800ng of pGIPZ shRNAmir construct. Cells transfected with pGIPZ shRNAmir constructs were imaged 72h after transfection and 4h after replating the cells. At least three different shRNA constructs were tested for each gene. Non-silencing pGIPZ shRNAmir (Cat no: RHS4346, Open Biosystems) was used as a negative control.

Generation of stable knock-down lines: To obtain stable knock-down cell lines, M2 cells stably expressing LifeAct-Ruby or GFP-Actin were transfected with the appropriate shRNA plasmids that had been linearised by digestion with *Ssp*I and selected with 250ng/ml puromycin for two weeks. Following selection, at least 90% of cells expressed the fluorescent protein reporter contained on the pGIPZ vector. These stable lines were then assayed for mRNA transcript expression by qPCR and for protein expression by Western blotting.

Phenotypic screening: To quantify the impact of stable gene depletion, cells stably transfected with targeting or non-silencing shRNA constructs were imaged and categorised as having normal, large, or small blebs. Expression of shRNA was verified on the basis of their expression of a fluorescent reporter protein present on the pGIPZ vector. Generally, at least 90% of cells in a stable population expressed the fluorescent reporter protein. The effect of targeting shRNAs on cellular phenotype was assessed by comparing the experimentally observed number of cells in each category to that expected for control cells expressing non-silencing shRNA using Chi-square tests. Results were deemed significant for $p<0.01$ and are

summarized in **Table S1**. For each shRNA, we verified that stable transfection led to a significant reduction in target mRNA transcript levels using qPCR (**Fig. S2K**) and in protein levels using Western blotting.

Construct	Accession	Oligo ID	Sequence mature sense
**Daam1 shRNA 1**	**NM_014992**	**V2LHS_229651**	**CGCTTTCAGACATTAATTA**
**Daam1 shRNA 2**	**NM_014992**	**V3LHS_339677**	**TCACTGAACATGACATCCA**
**mDia1 shRNA 1**	**NM_001079812**	**V2LHS_43609**	**CCAATTCTGCTCATAGAAA**
mDia1 shRNA 2	NM_001079812	V2LHS_43611	GGATTAATTGATCAAATGA
mDia1 shRNA 3	NM_001079812	V3LHS_392378	CAGATAGTTCTGCACAAGA
mDia1 shRNA 4	NM_001079812	V3LHS_392377	AAGATGTTTCAGATGAACA
**Fhod1 shRNA 1**	**NM_013241**	**V3LHS_313962**	**ACGGTCACCCTCATCAACA**
Fhod1 shRNA 2	NM_013241	V2LHS_71583	CCTTCAAGCTGGACTATGA
Fhod1 shRNA 3	NM_013241	V2LHS_71587	CGTGCACCCAGGCTCTCTA
**Arp3 shRNA 1**	**NM_005721**	**V2LHS_5786**	**GAGCTAGTATCTTGGATTA**
**Arp2 shRNA 1**	**NM_005722**	**V3LHS_341062**	**TAACTTCTAACATTGATCG**
**Arp2 shRNA 2**	**NM_005722**	**V3LHS_341063**	**TTCTTGGTACTCTTGTCGG**

In the table, the name of the construct, the accession number for the gene, the manufacturer's oligo ID, and the mature sense sequence of the hairpin structure of the construct is described (Open Biosystems). shRNA constructs used to generate stable knock-down lines are shown in bold, while those used only in transient transfection experiments are shown in normal font.

siRNA transfection of HeLa cells

For acute knockdown of mDia1 and ARPC2, GFP-Actin HeLa were transfected by lipofection with Lipofectamine RNAiMAX (Invitrogen, Life Technologies). Cells were plated on glass-bottom imaging dishes (MatTek, Ashland, MA, USA) at least 16 hours prior to transfection. *DIAPH1* siRNA was purchased from Invitrogen (HSS102771, Invitrogen, Life technologies). *ARPC2* siRNAs were purchased from ThermoScientific (ON-TARGET plus Human ARPC2 10109, Thermoscientific, Waltham, MA, USA) and were identical in sequence to those used in [S22]. Stealth RNAi Negative Control Med GC (at a final concentration of 20nM, Invitrogen, Life Technologies) was used as a non-silencing control for knockdown experiments. Knock-down efficiency was verified by Western blotting. Cells depleted in mDia1 and ARPC2 were imaged starting 72 hours post transfection.

Confocal microscopy

All fluorescence imaging (except for laser ablation experiments and FRAP experiments) was performed using a 100× oil-immersion objective on an inverted microscope (IX81, Olympus) fitted with a spinning disk head (Yokogawa, CSU22). Images were acquired with an Andor iXon camera and analyzed using Metamorph (Molecular Devices), ImageJ (http://rsbweb.nih.gov/ij/), and Excel (Microsoft) software. Excitation with a 488nm wavelength laser was utilised for GFP-tagged proteins, with a 543nm wavelength laser for RFP-, Ruby-, and mCherry-tagged proteins as well as TRITC-labelled antibodies, and with a 405nm wavelength laser for BFP-tagged shRNAs.

Long term time-lapse imaging

To assess the impact of mDia1 depletion and Arp2/3 inhibition/depletion on cell morphogenesis during the cell cycle, cells were examined using long-term time-lapse imaging.

To increase the proportion of mitotic cells, cells were first arrested in S phase with a single thymidine block by treatment with 2mM thymidine for ~16-24 hours. The block was released by replacing thymidine containing medium with normal DMEM medium ~7-8 hours before the start of imaging. CK666 (Tocris Bioscience) was used at 100µM. For time lapse experiments, the cells were plated on glass-bottom dishes (MatTek), maintained at 37 °C and supplied with 5% CO2 on the microscope stage. Multiposition long term time lapse microscopy was performed on a Zeiss axiovert 200M time lapse microscope using a 20X air objective (numerical aperture = 0.5). In some experiments, to image the impact of mDia1 depletion and/or Arp2/3 depletion/inhibition on the actin cortex, fluorescence microscopy of HeLa GFP-Actin cells was performed on Leica TCS SP5 confocal microscope using a 63X oil objective. All experiments were performed in triplicate. For high resolution imaging, images of at least 10 different cells were acquired for each experimental condition.

Fluorescence Recovery After Photobleaching (FRAP)
FRAP experiments were performed using a 100x oil immersion objective lens (NA=1.3, Olympus) on a scanning laser confocal microscope (Olympus Fluoview FV1000; Olympus). GFP-tagged proteins were excited with a 488nm wavelength laser. Fluorescence recovery of GFP-actin, GFP-CA-mDia1, GFP-Daam1 and GFP-GBD+FH3 was monitored over a small circular area (r=2µm) and bleaching was performed on a circular bleach spot (r=1µm) in the centre of the imaging region. This setup helped minimise loss of fluorescence through imaging in the sample. Bleaching was carried out by scanning the 488nm laser on full power line by line over the bleach region. The FRAP protocol was the following: first, five frames were acquired for normalisation of the fluorescence signal, then, the fluorescence was bleached with a single iteration of the bleach pulse at 8µs/pixel, and finally recovery was imaged over 100 frames acquired at 1s intervals. Graphs displaying fluorescence intensity normalised to the initial fluorescence intensity were output using the Fluoview software and analyzed using Origin software (OriginLab, Northampton, MA). FRAP experiments were performed on at least 14 different cells from at least three independent dishes.

Drug treatments
Cytochalasin D (Merck Biosciences, Darmstadt, Germany), a small molecule that promotes actin depolymerisation by capping the fast growing end of F-actin filaments was used in M2 cells stably expressing actin-GFP at 40nM (**Fig S2J**) and at 5µM in M2 cells stably expressing LifeAct-Ruby transfected with CA-mDia1-GFP (**Fig S3A**). CK666 (Tocris, Bristol, UK), a small molecule inhibitor against the Arp2/3 complex [S23], was used to study the role of the Arp2/3 complex in the cortex. In all cases, cells were first imaged for 24 time points at 10s intervals to provide baseline behaviour for comparison, then 100µM CK666 was added and incubated for 3 min at room temperature. During this time, the microscope was refocused choosing a plane that cut through the centre of the nucleus because CK666 caused rounding of the cells. After 3 min incubation the cells were imaged for an additional 35 time points. Other work in our laboratory has confirmed the efficacy of CK666 in inhibiting the Arp2/3 complex [S24, 25].

For all drugs, an equal amount of DMSO was used as a vehicle control. All imaging was performed on at least three separate experimental days.

RNA extraction and Quantitative Real-Time PCR
Total RNA from M2 and HeLa cells was extracted using the RNeasy Mini Kit (Qiagen, Hilden, Germany) and reverse transcribed using the High Capacity cDNA Reverse Transcription Kit (Applied Biosystems, Carlsbad, CA) following manufacturer protocols. The gene expression

level for endogenous controls *GAPDH* (Hs00266705) and *ACTB* (Hs00357333) was determined using pre-validated Taqman Gene Expression Assays (Applied Biosystems), and gene expression level for genes of interest was determined using assays designed with the Universal Probe Library (UPL) from Roche (www.universalprobelibrary.com) according to manufacturer's instructions. Sequences of primers used are available upon request.

Scanning electron microscopy

Sample preparation for scanning electron microscopy was performed as described in [S26] with minor modifications. Two hours prior to sample preparation, whole cells were plated onto 12mm glass coverslips. Immediately prior to fixation, the coverslips were washed three times with intracellular buffer (for isolated blebs) or L15 without serum (for cells) and transferred to cytoskeleton buffer (50mM Imidazole, 50mM KCl, 0.5mM $MgCl_2$, 0.1mM EDTA, 1mM EGTA, pH 6.8) containing 0.5% Triton-X and 0.25% glutaraldehyde for 5 min. This was followed by a second extraction with 2% Triton-X and 1% CHAPS in cytoskeleton buffer for 5 min before washing the coverslips in cytoskeleton buffer three times. The remainder of the protocol was identical to [S26]. The cells were then dehydrated with serial ethanol dilutions, dried in a critical point dryer, coated with 5-6nm platinum-palladium and imaged using the in-lens detector of a JEOL7401 Field Emission Scanning Electron Microscope (JEOL, Tokyo, Japan). All samples were prepared in duplicate and images from two separate experimental days were acquired. Similar phenotypes were observed on both experimental days and images of at least 10 different cells were acquired for each experimental condition.

Imaging screen

To assess the localisation of nucleators in cells, we used the following imaging procedures sequentially until we were able to resolve localisation: i) transfection of GFP-tagged full length proteins, ii) simultaneous permeabilization and fixation of cells transfected with GFP-tagged full length constructs, iii) immunostaining when antibodies were available.

Immunostaining

For immunostaining, cells were fixed with 2% formaldehyde and 0.1% glutaraldehyde with 0.2% Triton X-100 in cytoskeleton buffer (50mM Imidazole, 50mM KCl, 0.5mM $MgCl_2$, 0.1mM EDTA, 1mM EGTA, pH 6.8) for 15 min at room temperature, washed three times with PBS supplemented with 10 mg/ml bovine serum albumin (BSA). Non-specific binding was blocked by incubation in PBS supplemented with BSA for 10min. The cells were then incubated for 60 min at room temperature with a polyclonal rabbit anti-p34-Arc/ARPC2 antibody (1:50 dilution, #07-227, Millipore) or a polyclonal rabbit anti-mDia1 antibody (1:50 dilution, AbCAM, ab96784) diluted in PBS with 1% BSA. Cells were then washed three times with PBS/BSA and incubated with 1:100 Goat anti-rabbit IgG Alexa 647 secondary antibody (Molecular Probes, Invitrogen) and 1:200 Alexa568-phalloidin (Invitrogen) in 1% BSA in PBS for 60 min at room temperature. Finally the sample was washed four times with PBS/BSA. All samples were prepared in triplicate and images from two separate experimental days were acquired.

Permeabilization-fixation for localisation of constructs in cells expressing GFP-tagged proteins

To remove fluorescence background due to cytoplasmic unbound proteins, we used a permeabilization-fixation approach. Permeabilization-fixation of cells was performed by replacing L15-FBS with 0.25% glutaraldehyde (Fluka), 0.5% Triton X-100 (Sigma), in cytoskeleton buffer (50mM Imidazole, 50mM KCl, 0.5mM $MgCl_2$, 0.1mM EDTA, 1mM EGTA, pH 6.8) during imaging.

Rhodamine-phalloidin microinjections

The day prior to microinjection, M2 cells were plated onto glass bottom petri dishes (Intracell, UK). For microinjection, borosilicate glass capillaries were pulled using a Sutter P-97 pipette puller (Sutter, CA) to obtain pipettes with a sharp tip of ~0.5µm diameter. Rhodamine-phalloidin (Life technologies, UK) was diluted to a final concentration of 20µg/ml in microinjection buffer (50mM K-glutamate, 0.5mM $MgCl_2$, pH 7.0) and centrifuged at top speed in a tabletop centrifuge to remove aggregates. Cells were microinjected with a Narishige IM300 microinjector (Narishige, Japan) using 0.5psi backpressure. Microinjections were done using a 40x objective. After microinjection, the cells were left to recover for at least 10 min. Live cells that had been properly microinjected were identified by their fluorescence. Timelapse videos of the cells were acquired over a period of 5 min using a 100× objective exciting the fluorophore with a 568nm laser and collecting emission at 610nm. Experiments were performed on at least three independent days.

Western blotting

For Western-blotting, cells transiently transfected with shRNA were FACS-sorted based on their GFP- or BFP-expression. Cells stably expressing shRNA were used without FACs sorting. Cells were detached by tripsinisation, spun down at 1200x*g* (HeLa cells) or 1000x*g* (M2 cells) for 3 min. The cell pellet was resuspended in 500µl D-PBS and kept on ice for 3 to 5 min. Cells were spun down at 8000x*g* for 5 min at 4°C. The supernatant was discarded. Equal volumes of D-PBS and 2x Laemmli Buffer were added to the cell pellet to a final concentration of 10000 cells per 10µl. Samples were prepared for SDS-PAGE by boiling them (at 95°C) for 5 min. The level of protein depletion was assessed by Western blot analysis. Anti-mDia1 antibodies were obtained from SIGMA or AbCAM, anti-Daam1 antibodies were obtained from AbCAM (ab56951, Cambridge, UK), anti-ARPC2 was obtained from Millipore (#07-227), anti-Arp2 (10125) was from SantaCruz Biotechnology, anti-Arp3 was from Cell Signalling (#4738), anti-GAPDH and anti-FHOD1 (NBP 83900) were from Novus Biologicals. All secondary antibodies used were from Jackson ImmunoResearch. Equal amounts of cell lysates were loaded on a polyacrylamide gel. For FACs sorted cells, we used 10000 FACS sorted cells per sample in 10µl. Proteins separated on SDS-PAGE were transferred onto a PVDF membrane (pore size 0.45µm). Membranes were blocked with 5% non-fat dry milk in TBS-T for 1 h at room temperature and subsequently incubated overnight (at 4°C) with the appropriate primary antibodies (rabbit anti-mDia1 dilution 1:200, goat anti-Arp2 dilution 1:1000, anti-ARPC2 dilution 1:500, rabbit anti-Arp3 dilution 1:500, rabbit anti-Fhod1 dilution 1:1000, mouse anti-Daam1 dilution 1:50000, mouse anti-GAPDH dilution 1:100000). After extensive washing with TBS-T, membranes were incubated with the according HRP-coupled secondary antibodies (dilution 1:10000) for 1 h at room temperature. After extensive washing with TBS-T, protein bands were visualised using ECL Detection kit (GE Healthcare) or SuperSignal West Femto (Thermo Scientific). For reprobing western blots the membranes were incubated in Stripping buffer (2% SDS, 50mM Tris-HCl pH 6.8, 100mM β-Mercaptoethanol) for 1 h at 55°C and extensively washed with TBS-T. All Western blotting was performed in duplicate.

Localisation of actin cortex regrowth in blebs

M2 LifeAct-Ruby cells were imaged for 4 min at 200ms intervals and fluorescence intensity was measured along the periphery of blebs after the end of expansion. To do this, a region was drawn along the fully expanded bleb perimeter in Metamorph and the fluorescence intensity was monitored until a fully formed cortex was observed (on average 40 s). Kymographs of the intensity changes were created and these were displayed in pseudocolours to aid determination of sites of cortex regrowth. In the pseudocolour images, cold colours represented low intensities

and warm colours high intensities. Altogether 20 blebs from at least three independent days were analysed, and one kymograph is presented in **Fig. S1B**. A similar analysis was performed for HeLa cells in which blebs had been generated by laser ablation (see below). An example is presented in **Fig. S1C**.

Quantum-dot phalloidin preparation and microinjection

We prepared quantum dot-phalloidin according to a protocol published by the manufacturer (Life technologies). Briefly, 2nM of quantum dots (Q-dots 565 ITK Amino PEG, Life Technologies) in 50mM borate buffer pH 8.3 were washed by ultrafiltration (using a 100kDa cut-off membrane) with 1xPBS pH 7.4. Then Q-dots were transferred into a glass vial and crosslinked to amino-phalloidin (Alexis chemicals) with Bis[sulfosuccinimidyl] suberate (BS3, Pierce) according to manufacturer's instructions. Finally, phalloidin functionalised Q-dots were filtered with a 0.22μm spin filter (Agilent technologies) and stored at 4°C. Microinjections were performed as described in Rhodamine-phalloidin microinjections paragraph. The Q-dot-phalloidin solution was diluted 1:30 in microinjection buffer and centrifuged at top speed in a tabletop centrifuge to remove aggregates. Timelapse videos of the cells were acquired over a period of 10-60min after microinjection, using a 100× objective exciting Qdots with a 488nm laser and collecting emission above 565nm. Experiments were performed on more than three independent days and presented the same phenotype on each day.

Generation of covalently stabilized fluorescent filaments

Actin was purified from rabbit muscle following established protocols [S27] and labelled with Alexa488 succinimidyl ester (Molecular Probes Invitrogen). The labelling fraction was 49% in our experiments. To polymerise actin filaments, 5μM labelled G-actin was incubated in F-buffer (20mM Tris pH 7.5, 0.2mM DTT, 0.2mM $CaCl_2$, 20mM $MgCl_2$, 10mM ATP, 1M KCl) for 30 min at room temperature. Then, p-NN'-phenylenebismaleimide (pPDM) (Sigma) was added up to a final concentration of 1mM to form covalent crosslinks between actin protomers in the filament [S5]. Stabilisation by pPDM was allowed to take place for 30 min at room temperature. Finally the sample was sonicated thoroughly and stored at 4°C until microinjections were performed. The size distribution of covalently crosslinked protomers within the filaments was assessed by 10% SDS-PAGE (**Fig. S1G**). Microinjections were performed as described in Rhodamine-phalloidin microinjections paragraph. Prior to microinjections the filaments were diluted 1:1 into microinjection buffer. Experiments were performed on more than three independent days and presented the same phenotype on each day.

Quantification of cortical actin fluorescence in M2 cells in live experiments

To assess loss of cortical actin induced by CK666 treatment or DMSO, we measured the average intensity of the cortex and the cytoplasm pre and post treatment. The intensity of the cytoplasm was measured in three different sites of the cell body excluding the area on top of nucleus. The average ratio and standard error of the mean were calculated prior to and after treatment. In images of cells expressing GFP-actin, cortical actin fluorescence results both from GFP-actin incorporated in filaments within the cortex and free monomeric GFP-actin diffusing through the cortical mesh. Hence, normalised cortical F-actin fluorescence can be estimated as $f=(F_{cortex}-F_{cytoplasm})/F_{cytoplasm}$ with F_{cortex} and $F_{cytoplasm}$ the mean fluorescence intensities of the cortex and cytoplasm respectively. Finally, t-tests were performed to assess statistical significance. Samples were prepared in duplicate and grouped for analysis.

Quantification of cortical F-actin fluorescence versus cytoplasmic F-actin fluorescence in phalloidin stained samples

To investigate what portion of the total cellular F-actin the submembranous cortex represents, we analysed fluorescence images of M2 blebbing melanoma cells stained with Alexa568-phalloidin. M2 cells have a well-defined actin cortex with few stress fibres, filopodia or microvilli and their cell body presents a very rounded morphology when examined within a few hours of plating. For our analysis, we acquired images of M2 cells through their equator. The actin cortex could then be segmented from the cytoplasm based on intensity with high intensity structures belonging to the cortex and low intensity structures part of the cytoplasm (Metamorph, Molecular Devices) (**Fig. S3J**). Following segmentation, the respective integrated fluorescence intensity of the whole cell and the actin cortex were measured and exported to Excel (Microsoft) for analysis.

Actin regrowth speed analysis

The rate of actin accumulation during cortex regrowth was measured as detailed in [S19]. To allow for reliable segmentation of the cell contour for subsequent image analysis, we added 2.5µM Alexa568 dye to the extracellular medium. Images were then processed, automatically analysed and visualised using a custom software package called KoreTechs, developed in MATLAB (MathWorks). The software, its source code and an exhaustive user guide are freely available online [S19]. Briefly, segmentation and tracking of cells was based on a combination of image filtering, histogram inversion and thresholding of raw fluorescent intensities of a non-permeable extra-cellular fluorescent dye. The cortex was then defined as a region of fixed depth underlying the segmented cell contour. The cytoplasmic region was taken as the difference between the segmented cell area and the cortical region. Bleb locations were automatically determined using local calculations of the osculating circles, and determining the two positions along the cell contour with highest positive curvature. The bleb region could thus be segmented from the cell body region and cortical and cytoplasmic fluorescence intensities of actin could then be monitored in the bleb and compared to the cell body control value. This analysis yielded curves relating the evolution of the actin fluorescence intensity in the bleb normalised to the cortical fluorescence intensity in the cell body. Actin accumulation in the cortex displayed two markedly different phases: the first started immediately after laser ablation and ended shortly after the cessation of growth; the second started after growth had finished and ended after bleb retraction (**Fig. 3J**). Actin accumulation was approximately linear in both regimes and slopes relating the percentage actin accumulation per second could be measured by fitting straight lines to each interval.

Statistical analysis

Phenotype distribution after gene depletion was compared to cells stably expressing non-silencing shRNA using a Chi-square test. Cells were imaged on at least two separate days. Values of $p<0.01$ were deemed statistically significant.

Changes in fluorescence recovery half times and ratio of cortex intensity to cytoplasmic intensity were compared across conditions using a Student t-test. Values of $p<0.01$ were deemed statistically significant.

Changes in actin accumulation rates across conditions were examined using a Mann-Whitney U-test. Values of $p<0.05$ were deemed statistically significant.

Changes in F-actin fluorescence intensity across conditions measured by flow cytometry were compared to a mean of 0 (corresponding to no change in F-actin intensity) with a one-way Anova test with Bonferroni post-test. Values of $p<0.05$ were deemed statistically significant due to the low number of repeats (3).

Supplemental References

S1. Charras, G.T., Hu, C.K., Coughlin, M., and Mitchison, T.J. (2006). Reassembly of contractile actin cortex in cell blebs. J Cell Biol *175*, 477-490.

S2. Alsop, G.B., Chen, W., Foss, M., Tseng, K.F., and Zhang, D. (2009). Redistribution of actin during assembly and reassembly of the contractile ring in grasshopper spermatocytes. PLoS One *4*, e4892.

S3. Charras, G.T., Mitchison, T.J., and Mahadevan, L. (2009). Animal cell hydraulics. Journal of cell science *122*, 3233-3241.

S4. Moeendarbary, E., Valon, L., Fritzsche, M., Harris, A.R., Moulding, D.A., Thrasher, A.J., Stride, E., Mahadevan, L., and Charras, G.T. (2013). The cytoplasm of living cells behaves as a poroelastic material. Nature materials.

S5. Knight, P., and Offer, G. (1978). p-NN'-phenylenebismaleimide, a specific cross-linking agent for F-actin. The Biochemical journal *175*, 1023-1032.

S6. Niedermayer, T., Jegou, A., Chieze, L., Guichard, B., Helfer, E., Romet-Lemonne, G., Carlier, M.F., and Lipowsky, R. (2012). Intermittent depolymerization of actin filaments is caused by photo-induced dimerization of actin protomers. Proceedings of the National Academy of Sciences of the United States of America *109*, 10769-10774.

S7. Gilbert, H.R., and Frieden, C. (1983). Preparation, purification and properties of a crosslinked trimer of G-actin. Biochem Biophys Res Commun *111*, 404-408.

S8. Doi, Y. (1992). Interaction of gelsolin with covalently cross-linked actin dimer. Biochemistry *31*, 10061-10069.

S9. Kim, E., Bobkova, E., Hegyi, G., Muhlrad, A., and Reisler, E. (2002). Actin cross-linking and inhibition of the actomyosin motor. Biochemistry *41*, 86-93.

S10. Cunningham, C.C. (1995). Actin polymerization and intracellular solvent flow in cell surface blebbing. J Cell Biol *129*, 1589-1599.

S11. Sanders, M.C., and Wang, Y.L. (1990). Exogenous nucleation sites fail to induce detectable polymerization of actin in living cells. The Journal of cell biology *110*, 359-365.

S12. Chhabra, E.S., Ramabhadran, V., Gerber, S.A., and Higgs, H.N. (2009). INF2 is an endoplasmic reticulum-associated formin protein. Journal of cell science *122*, 1430-1440.

S13. Cunningham, C.C., Gorlin, J.B., Kwiatkowski, D.J., Hartwig, J.H., Janmey, P.A., Byers, H.R., and Stossel, T.P. (1992). Actin-binding protein requirement for cortical stability and efficient locomotion. Science *255*, 325-327.

S14. Ory, D.S., Neugeboren, B.A., and Mulligan, R.C. (1996). A stable human-derived packaging cell line for production of high titer retrovirus/vesicular stomatitis virus G pseudotypes. Proceedings of the National Academy of Sciences of the United States of America *93*, 11400-11406.

S15. Lagal, V., Abrivard, M., Gonzalez, V., Perazzi, A., Popli, S., Verzeroli, E., and Tardieux, I. (2014). Spire-1 contributes to the invadosome and its associated invasive properties. Journal of cell science *127*, 328-340.

S16. Gill, M.B., Roecklein-Canfield, J., Sage, D.R., Zambela-Soediono, M., Longtine, N., Uknis, M., and Fingeroth, J.D. (2004). EBV attachment stimulates FHOS/FHOD1 redistribution and co-aggregation with CD21: formin interactions with the cytoplasmic domain of human CD21. Journal of cell science *117*, 2709-2720.

S17. Watanabe, N., and Mitchison, T.J. (2002). Single-molecule speckle analysis of actin filament turnover in lamellipodia. Science *295*, 1083-1086.

S18. Ai, H.W., Shaner, N.C., Cheng, Z., Tsien, R.Y., and Campbell, R.E. (2007). Exploration of new chromophore structures leads to the identification of improved blue fluorescent proteins. Biochemistry *46*, 5904-5910.

S19. Biro, M., Romeo, Y., Kroschwald, S., Bovellan, M., Boden, A., Tcherkezian, J., Roux, P.P., Charras, G., and Paluch, E.K. (2013). Cell cortex composition and homeostasis resolved by integrating proteomics and quantitative imaging. Cytoskeleton (Hoboken) *70*, 741-754.

S20. Carriere, A., Cargnello, M., Julien, L.A., Gao, H., Bonneil, E., Thibault, P., and Roux, P.P. (2008). Oncogenic MAPK signaling stimulates mTORC1 activity by promoting RSK-mediated raptor phosphorylation. Current biology : CB *18*, 1269-1277.

S21. Rappsilber, J., Ryder, U., Lamond, A.I., and Mann, M. (2002). Large-scale proteomic analysis of the human spliceosome. Genome Res *12*, 1231-1245.

S22. Derivery, E., Fink, J., Martin, D., Houdusse, A., Piel, M., Stradal, T.E., Louvard, D., and Gautreau, A. (2008). Free Brick1 is a trimeric precursor in the assembly of a functional wave complex. PLoS One *3*, e2462.

S23. Nolen, B.J., Tomasevic, N., Russell, A., Pierce, D.W., Jia, Z., McCormick, C.D., Hartman, J., Sakowicz, R., and Pollard, T.D. (2009). Characterization of two classes of small molecule inhibitors of Arp2/3 complex. Nature *460*, 1031-1034.

S24. Bergert, M., Chandradoss, S.D., Desai, R.A., and Paluch, E. (2012). Cell mechanics control rapid transitions between blebs and lamellipodia during migration. Proceedings of the National Academy of Sciences of the United States of America *109*, 14434-14439.

S25. Wilson, K., Lewalle, A., Fritzsche, M., Thorogate, R., Duke, T., and Charras, G. (2013). Mechanisms of leading edge protrusion in interstitial migration. Nat Commun *4*, 2896.

S26. Svitkina, T.M., and Borisy, G.G. (1998). Correlative light and electron microscopy of the cytoskeleton of cultured cells. Methods Enzymol *298*, 570-592.

S27. Spudich, J.A., and Watt, S. (1971). The regulation of rabbit skeletal muscle contraction. I. Biochemical studies of the interaction of the tropomyosin-troponin complex with actin and the proteolytic fragments of myosin. The Journal of biological chemistry *246*, 4866-4871.
