# Supplementary figures and images for: Cellular Control of Cortical Actin Nucleation
Source: Curr Biol. 2014 Jul 21;24(14):1628–35. doi: 10.1016/j.cub.2014.05.069 (PMC4110400; doi:10.1016/j.cub.2014.05.069)

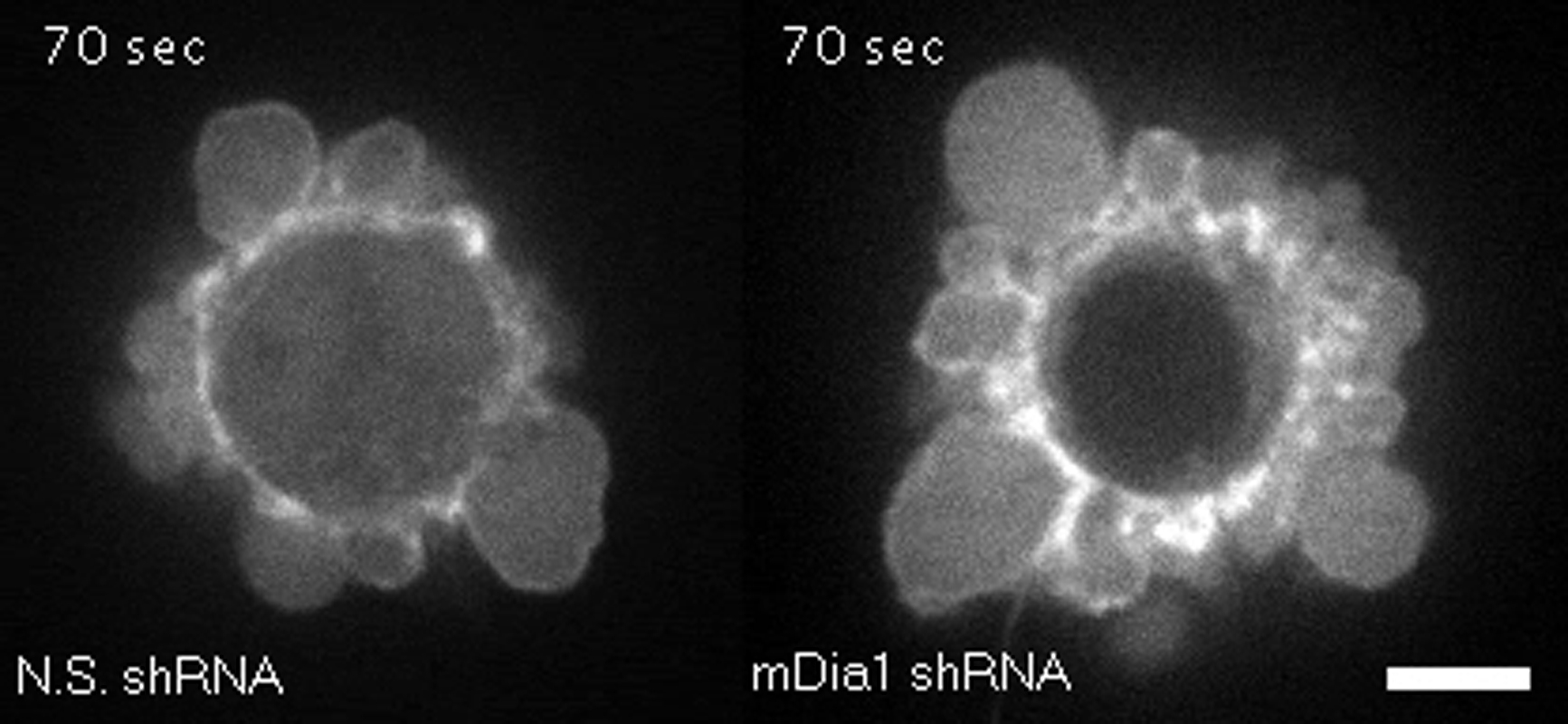

Supplement: Movie S1. Representative Blebbing Cells Expressing Nonsilencing shRNA and shRNA Targeting mDia1 — M2 blebbing cells stably expressing GFP-actin transfected with nonsilencing shRNA (left) or shRNA targeting mDia1 (right). Depletion of mDia1 (right) led to the formation of very large blebs compared to control cells (left), but the cells still retained a well-defined actin cortex. Transfected cells were identified based on the expression of a BFP marker present on the shRNA vector. Scale bar represents 5 μm. Total duration is 230 s. [file mmc2.jpg]

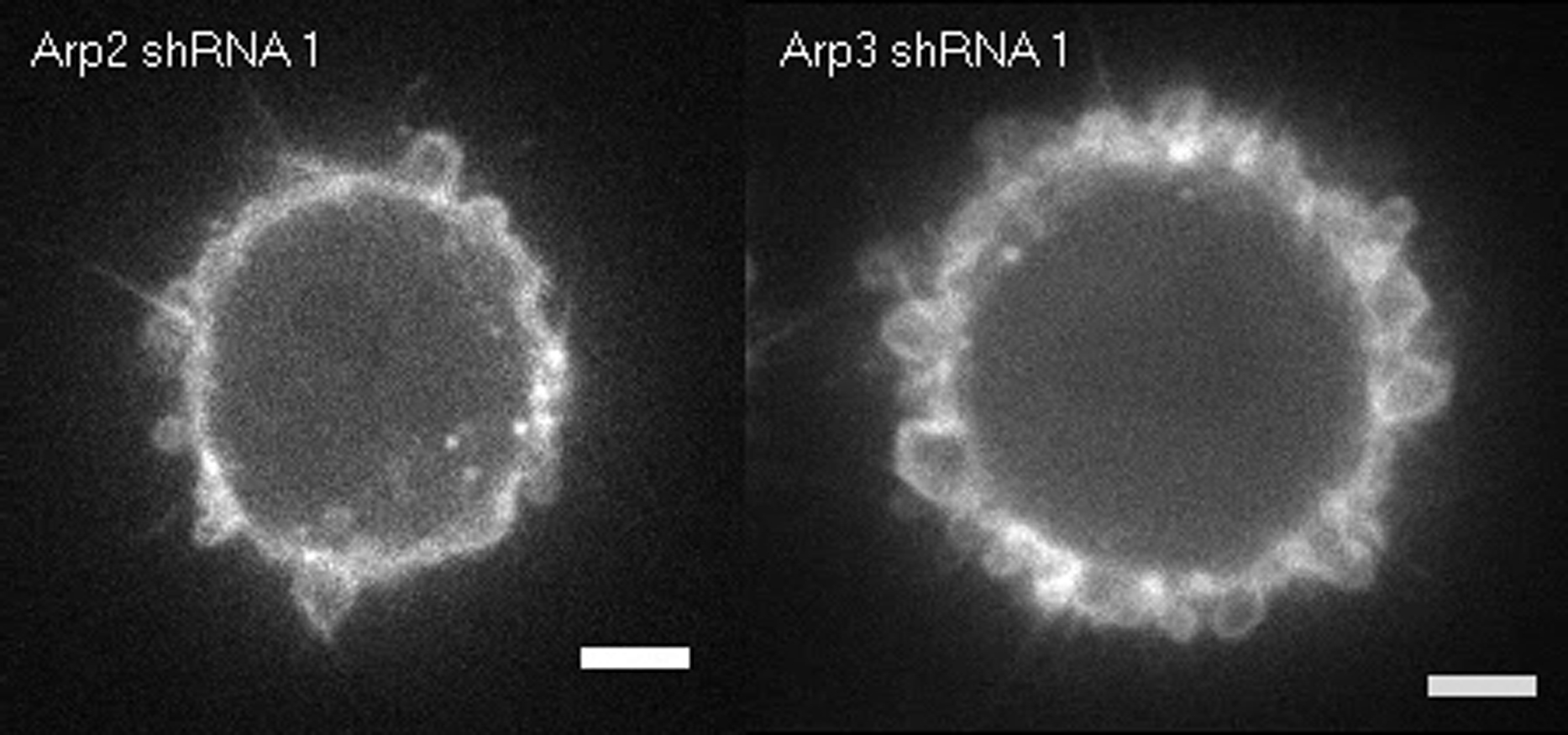

Supplement: Movie S2. Representative Blebbing Cells Expressing shRNA Targeting Arp2 and Arp3 — M2 blebbing cells stably expressing LifeAct Ruby and stably transfected with shRNA targeting Arp2 (left) or shRNA targeting Arp3 (right). Depletion of Arp2 or Arp3 led to the formation of small blebs compared to control cells (Movie S1, left panel). Transfected cells were identified based on the expression of a GFP marker present on the shRNA vector. Scale bar represents 3 μm. Total duration is 600 s. [file mmc3.jpg]

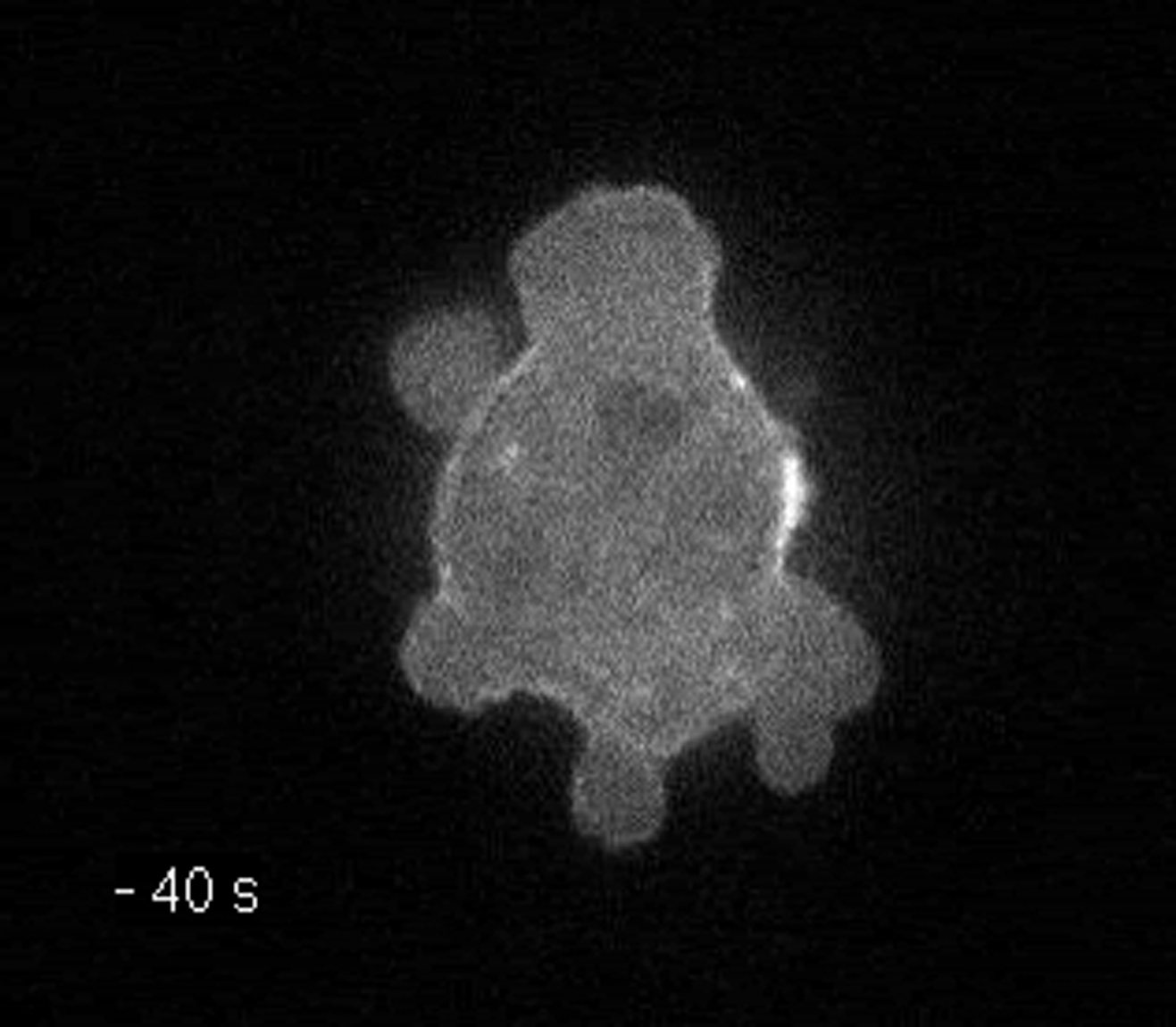

Supplement: Movie S3. Representative Blebbing Cell Depleted in mDia1 prior to and during Treatment with Arp2/3 Complex Inhibitor — M2 blebbing cell stably expressing GFP-actin transfected with shRNA targeting mDia1. Prior to treatment, the cell formed large blebs and retained a clear actin cortex. After treatment, the cell rapidly lost its shape and the majority of its cortical actin. The cell only retained a few discernible foci of cortical actin, and the nucleus was expelled from the cell body into a large bulge. Transfected cells were identified based on the expression of a BFP marker present on the shRNA vector. The timing of addition of CK666 is indicated on the movie. A 300 s pause occurred after addition of CK666 to allow for refocusing due to cell rounding in response to treatment. Images during this pause are not included in the movie. Scale bar represents 5 μm. [file mmc4.jpg]
